# Comparative analysis of diagnostic ultrasound and histopathology for detecting cervical lymph node metastases in head and neck cancer

**DOI:** 10.1007/s00432-023-05439-x

**Published:** 2023-10-12

**Authors:** Karl Christoph Sproll, Iryna Hermes, Gerd Felder, Nikolas H. Stoecklein, Maximilian Seidl, Peter Kaiser, Wolfgang Kaisers

**Affiliations:** 1 Department of Oral and Maxillofacial Surgery, Medical Faculty and University Hospital Düsseldorf, Heinrich-Heine-University Düsseldorf, Moorenstraße 5, 40225 Düsseldorf, Germany; 2 Coordination Center for Clinical Trials, Medical Faculty and University Hospital Düsseldorf, Heinrich-Heine-University Düsseldorf, Düsseldorf, Germany; 3 Department of General, Visceral and Pediatric Surgery, Medical Faculty and University Hospital Düsseldorf, Heinrich-Heine-University Düsseldorf, Düsseldorf, Germany; 4 Department of Pathology, Medical Faculty and University Hospital, Düsseldorf, Heinrich-Heine-University Düsseldorf, Düsseldorf, Germany; 5 Institute for Pathology, Dermatopathology, Cytology and Molecular Pathology, Wetzlar, Germany; 6 Department of Anesthesiology, Sana Hospital Benrath, Düsseldorf, Germany

**Keywords:** Diagnostic ultrasound, Lymph node metastasis, Roundness

## Abstract

**Purpose:**

We evaluated the current performance of diagnostic ultrasound (US) for detecting cervical lymph node (LN) metastases based on objective measures and subjective findings in comparison to the gold standard, histopathological evaluation.

**Patients and methods:**

From 2007 to 2016, we prospectively included patients with head and neck cancer who were scheduled for surgical therapy including neck dissection. LNs were examined by multimodal US by a level III head and neck sonologist and individually assigned to a map containing six AAO-HNS neck LN levels preoperatively. During the operation, LNs were dissected and then assessed by routine histopathology, with 86% of them examined individually and the remaining LNs (14%) per AAO-HNS neck LN level. The optimal cutoff points (OCPs) of four defined LN diameters and 2D and 3D roundness indices per AAO-HNS neck LN level were determined.

**Results:**

In total, 235 patients were included, and 4539 LNs were analyzed by US, 7237 by histopathology and 2684 by both methods. Of these, 259 (9.65%) were classified as suspicious for metastasis by US, whereas 299 (11.14%) were found to be positive by histopathology. Subjective US sensitivity and specificity were 0.79 and 0.99, respectively. The OCPs of the individual LN diameters and the 2D and 3D roundness index were determined individually for all AAO-HNS neck LN levels. Across all levels, the OCP for the 2D index was 1.79 and the 3D index was 14.97. The predictive performance of all distances, indices, and subjective findings improved with increasing metastasis size. Anticipation of pN stage was best achieved with subjective US findings and the smallest diameter (Cohen’s κ = 0.713 and 0.438, respectively).

**Conclusion:**

Our LN mapping and meticulous 1:1 node-by-node comparison reveals the usefulness of US for detecting metastatic involvement of neck LNs in head and neck carcinomas as compared to histopathology. The predictive ability for small tumor deposits less than 8 mm in size remains weak and urgently needs improvement.

**Supplementary Information:**

The online version contains supplementary material available at 10.1007/s00432-023-05439-x.

## Introduction

Metastasis to the cervical lymph nodes (LNs) is the single most relevant prognostic factor for patients suffering from head and neck cancer (Gil et al. 2009). Neck dissection (ND) and histopathological examination of excised LNs is currently the gold standard for diagnosing LN involvement even in early tumor stages (de Bree et al. 2015). Despite its potentially serious side effects (nerve injury, scarring, constrictions), elective ND in its various scopes, therefore, remains a cornerstone in the surgical treatment of epithelial malignancies of the upper aerodigestive tract (D’Cruz et al. 2015). To date, no routine or study-tested method has a diagnostic accuracy comparable to that of histopathologic examination.

Ultrasound (US) is useful for LN diagnosis because it is a rapid, noninvasive, and cost-effective method to identify LNs and assess their size, shape, and consistency (Ahuja and Ying 2005). The superficial locations of cervical LNs make them particularly suitable for examination by high-frequency B-scan US, and it provides valuable diagnostic information on the internal architecture and intranodal blood flow of these LNs (de Bree et al. 2015). Some of the limitations of US are related to examiner dependence, weakness in detecting small tumor nests, and lack of specificity (Leusink et al. 2012). Previous studies on the same subject are in part much older and have, therefore, not yet been able to take into account the technical developments of recent years. One of them was retrospective and used only FNAB of representative LNs as a reference (Ying et al. 1999). Another one used only histologic correlation per neck level (except IIB) (Hohlweg-Majert et al. 2009), and the third one summarily compared US and pathologic findings per patient (Hajek et al. 1986). Nevertheless, they report sensitivities of 74.1% and specificities of 91.5% (Hohlweg-Majert et al. 2009) or sensitivities of 75–98% and specificities of 41–88%, depending on LN neck level (Ying et al. 1999).

Prospective studies on the diagnostic power of neck US are lacking, and the capabilities of exact mapping based on AAO-HNS neck LN levels (Som et al. 2000) and of stringent assignment by means of sophisticated and standardized ND techniques have not yet been exhaustively explored.

The goal of any imaging procedure must be to compete with the gold standard, namely, histopathologic findings, to obviate the need for surgical staging in the future. In addition to avoiding surgical risks and operating time, the draining LNs should nowadays also be preserved for the effect of the emerging immunotherapeutic approaches (van Pul et al. 2021).

Therefore, the aim of the present study was to establish objective measures (e.g., LN length, width, and roundness) for performing US to detect metastases in cervical LNs and to validate these diagnostic criteria in addition to subjective findings, namely, the determinations made by the US specialist. For this, we first examined LNs of patients with Head and Neck Cancer by US and then assessed the same LNs upon meticulous 1:1 node-by-node comparison by histopathology, and correlated the results.

## Materials and methods

### Study design and patients

This noninterventional, prospective, monocentric clinical study included patients who were referred to our internal US department from 01/01/2007 to 06/15/2016. All patients included had a histologically confirmed carcinoma of the head and neck region or a relapse thereof and were scheduled for examination of the presence of cervical LN metastases by US. The obtained findings were to be correlated with the histopathologic results, requiring at least excision of one LN up to and including comprehensive ND as part of the diagnosis or treatment of the underlying disease.

Exclusion criteria have been: diagnosis other than head and neck cancer, inability to passively participate in US (duration 30–45 min), age less than 18 years, induction treatment by radiation or drug therapy and treatment without LN dissection.

We planned to enroll among the study cohort at least 200 patients with therapy-naïve primary head and neck squamous cell carcinoma (HNSCC) who were assigned for surgical therapy upon consultation of the multidisciplinary tumor board. The study protocol was reviewed and approved by the ethics committee of Heinrich-Heine-University Düsseldorf (# 4231). All patients provided written consent for the sampling of LNs and data collection.

### US examination of neck LNs

**Fig. 1:**
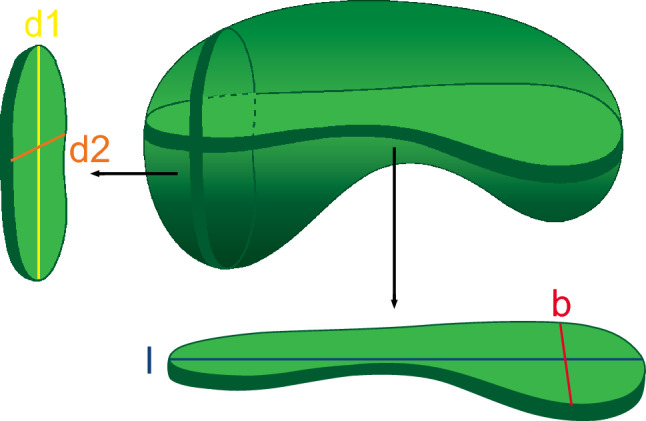
Schematic 3D representation of a bean-shaped LN. The distances measured by ultrasound were as follows: Longest Cross-Sectional Diameter (l), Longest Perpendicular Diameter (b), Longest Diameter Perpendicular to the Plane (d1), Longest Diameter Perpendicular to d1 (d2). A spherical, concentrically growing mass (metastasis) will initially lead to an increase in the smaller distances b and d2 and only secondarily to the distances d1 and l

US examinations were performed by KCS (level III sonologist for Head and Neck ultrasound of the German Society for Ultrasound in Medicine (DEGUM)) on the day before surgery using the Acuson Antares System Premium Edition and the VFX 13-5 Multi-D linear probe (both Siemens Medical Solutions®, Mountain View, CA, USA) on individually tailored preset “lymph nodes”, which had been further optimized for the individual AAO-HNS neck LN levels I, II, and III (Robbins et al. 2002), each as described previously (Heusch et al. 2014; Sproll et al. 2017; Sproll et al. 2021). In the ultrasound (US) examination, each lymph node (LN) was individually evaluated based on its imaging features and anatomical location. The measurements were carefully documented, capturing four specific distances to provide a comprehensive geometric description of the LN. The descriptions of the four distances measured are the following: Longest Cross-Sectional Diameter (l): This is the maximum distance measured across the lymph node in the plane that yields the largest cross-sectional area. Essentially, it is the longest axis that can be drawn through the lymph node in a single 2D plane. Longest Perpendicular Diameter (b): once the longest cross-sectional diameter (l) is determined, the longest perpendicular diameter (b) is the maximum distance measured at a 90° angle to “l” within the same 2D plane. Longest diameter perpendicular to the plane (d1): This distance is measured by identifying the longest diameter that lies perpendicular to the plane containing the longest cross-sectional diameter (l) and the longest perpendicular diameter (b). Longest diameter perpendicular to d1 (d2): After establishing the longest diameter perpendicular to the plane (d1), the distance d2 is the maximum length measured perpendicular to d1, essentially capturing the third dimension of the LN if one considers l and d1 as the other two dimensions. These measurements collectively aim to provide a multidimensional assessment of the LNs size and shape, assisting in further diagnostic evaluations (Fig. 1). The LN was then depicted schematically on a diagram containing the six AAO-HNS neck LN levels (Suppl. Fig. 1a) (Som et al. 1999, 2000). Additionally, subjective US findings were recorded for each LN. For more details on the subjective assessment of the US results, please refer to Suppl. Doc. 1.

The result of the subjective LN examination was an assessment of the LN as either containing metastasis or tumor-free. Patient treatment was carried out as described in Suppl. Doc. 1 (NCCN 2018; Wolff 2019).

### Preparation and histopathological assessment of dissected LNs

ND specimens were processed in the operating room to retrieve each LN identified by US, and each LN was recorded on the patient’s LN map, as described previously [Supporting information No. 1 in (Sproll et al. 2017)]. Each LN was then freed from surrounding fibrofatty tissue, placed in an individually labeled container with Formaldehyde Solution 4% phosphate buffered for histology (neoFroxx, Einhausen, Germany) and sent for routine histopathological assessment (Suppl. Fig. 2). The remaining ND specimen was dissected according to the AAO-HNS neck LN (sub) level (1–6) and assessed for each neck LN level by histopathology (Robbins et al. 2002). Details of histopathological evaluation are given in Suppl. Doc. 1. pTNM staging was performed according to the sixth and seventh editions of the TNM classification of malignant tumors, as amended at the time (Sobin et al. 2011). If metastases were found in the histopathological assessment, their maximum diameters (in mm) were measured in two planes.

### Data acquisition and processing

Study endpoints were the agreement of sonographic and histopathological findings as well as the optimal cutoff values for the individual diameters and the roundness indices in the prediction of metastatic disease. All patient-related data (clinical course, US and pathology) were stored in a special GCP-compliant individually designed database we have named “Lymphsono” based on the OmniComm® Electronic Data Capture Software broadly used in our study center (OmniComm Systems, Inc.; Fort Lauderdale, FL, USA) (Suppl. Fig. 3). Access was controlled to ensure that the data could be viewed and edited only by authorized personnel. The database has been continuously adapted and improved, and changes have been documented in the program history to keep a record of the development of the program.

### Statistical analysis

Count data are presented as the total number of LNs in the patient cohort and the mean and median (range) number of LNs per patient. Data were extracted from the OmniComm database, and all statistical calculations were executed in R (R Development Core Team 2020). Fisher’s exact test for contingency tables larger than 2 × 2 based on FEXACT (Mehta and Patel 1986) improved as described by Clarkson et al. (1993) was used. Two indices were used to describe roundness: the two-dimensional index (the Solbiati index), namely, 2D roundness = $\frac{l}{b}$ (Solbiati et al. 1992) (Fig. 1) and a three-dimensional index, namely, 3D roundness = $\left(\frac{l}{b}\right)$^2^ × $\left(\frac{l}{d1}\right)$^2^ × $\left(\frac{l}{d2}\right)$^2^. Both indices assume a value of 1 for a sphere and increase as l increases compared to the other distances, but the 3D index suggested a greater spread and may have better performance.

For analysis of count data, exact binomial tests and Fisher’s exact tests were employed (both using the statistical package). OCP analysis was carried out using the pROC package in R (Altman and Bland 1994a, b; Robin et al. 2011). The unweighted Cohen kappa index (κ) was calculated using the “irr” package in R (Cohen 1960).

For the subjective ultrasound findings, we calculated the sensitivity, specificity, positive, and negative predictive value (PPV and NPV, respectively) and likelihood ratio (LR) (Altman and Bland 1994a, b). For individual distances l, b, d1, and d2 (Fig. 1) and the two roundness indices, optimal cutoff points (OCPs) were determined by the maximal Youden index (YI) (*t*_Y_ = max_c_ (Se (c) + Sp (c) − 1)) for each individual neck level using the pROC package in R (Robin et al. 2011). The area under the curve (AUC), YI, sensitivity, specificity, PPV, and NPV were also determined using the OCP. Furthermore, the performance of the subjective ultrasound findings, the two roundness indices and the individual distances (l, b, d1, and d2) were determined depending on the sizes of the metastases detected by histopathological analysis (≤ 2 mm, > 2 and ≤ 4 mm, > 4 and ≤ 6 mm, > 6 and ≤ 8 mm, > 8 and ≤ 10 mm, and > 10 mm). On the basis of the OCPs of individual distances l, b, d1, and d2, the two indices for each individual neck level (1–6) (Robbins et al. 2002) and the subjective ultrasound findings for individual LNs, a clinical N stage (cN stage) was determined for each patient and compared to the pathologically determined N stage (pN stage). These classifications were carried out both for individual N stages (Sobin et al. 2011) and for the summary categories “metastasis present or not present” (N + vs. N0). Cohen’s kappa index was determined for each individual distance, the two roundness indices and the subjective ultrasound findings. The Landis and Koch classification was applied to systematize data with values between 0 (worthless) and 1 (perfect) (Landis and Koch 1977).

## Results

### General patient cohort

In total, 235 patients were enrolled, comprising 97 women (41.28%) and 138 men (58.72%). The average age at the time of tumor surgery was 64.9 years (median 66.0 years). 202 of them suffered from primary therapy-naïve HNSCC (lip mucosa to oropharynx), 3 from cervical CUP syndrome (2 of SCC and 1 of melanoma), 4 with verrucous carcinoma of the oral cavity, 1 with undifferentiated sinus carcinoma, 6 with salivary gland adenocarcinoma, 1 with carcinoma of the Meibomian glands, 1 with malignant melanoma, 1 with recurrence of mucoepidermoid carcinoma, 1 with recurrence of melanoma, and 15 with recurrence of HNSCC (lip mucosa to oropharynx). Among these patients, there were 248 surgical treatment sessions with varying extents of ND.

### Lymph node analysis by ultrasound and neck dissection

Overall, 4539 (1–42, mean 18.38, median 18 per patient) LNs were identified by US preoperatively. Of these, 265 (5.84%; 0–16, mean 1.07, median 0 per patient) of the identified LNs were classified as suspicious of malignancy according to subjective sonographic findings. The remaining LNs were categorized as reactively enlarged and tumor free. Based on our subjective assessment of LNs, each patient was assigned a cN status preoperatively.

During subsequent tumor surgery, 7237 (1–126, mean 29.30, median 24 per patient) LNs were removed from tissue obtained by ND and analyzed by histopathology. Among these, metastases were found in 443 LNs (6.12%; 0–57, mean 1.79, median 0 per patient). The pN classification was based on histopathological results and used as a reference for the abovementioned cN classification. A total of 2684 LNs were evaluated using both methods, and their results compared. Of the LNs evaluated by both methods, 2304 (85.8%) were identified individually from the dissected neck specimens in the operating room (OR), marked on the neck diagram of the corresponding patient analogously to the US findings and then sent in individual containers for 1:1 histopathological assessment (Suppl. Fig. 3). An additional 380 LNs (14.2%) were sent along with the specimens, which were still dissected in the OR, allotted according to the AAO-HNS neck LN level and analyzed separately per neck LN level (Robbins et al. 2002). Of the 2684 LNs, 259 (9.65%) were classified as suspicious for metastasis by US, but 299 (11.14%) were found to be positive by histopathology. Discordant findings were found in 86 cases (23 false positive (Suppl. Fig. 4a–c) and 63 false negative (Suppl. Fig. 5a–c) results by US). According to histopathology, pN status was pN0 in 133, pN1 in 41, pN2 in 2, pN2a in 3, pN2b in 45, pN2c in 19, and pN3 in 5 cases.

### Determination and predictive performance of optimal cutoff points (OCPs)

To maximize the performance of the objective criteria in predicting tumor involvement, the OCPs for individual diameters and indices per level (I–VI) and sublevel (IA–B, IIA–B, and VA–B) were determined by ROC analysis (Table 1). There was a clear spread among the values across neck LN levels. For example, the 2D roundness value for level IA was 1.5 and that for level III was 2.01. For diameter l, the OCP was 6.1 mm for level IV and 14.95 mm for level IIA. For individual diameters, there was a general spread from the smallest to largest value by a factor of two (Table 1). Determination of the individual OCPs per level reflects the shape variations of the LNs in the individual levels and led to an improvement in the prediction of metastatic disease.

**Table 1:** a Optimal cutoff points and performance of the 2D- and 3D-indices per AAO-AHNS neck LN level. b Optimal cutoff points (in mm) and performance of the individual distances per AAO-AHNS neck LN level. c Performance of the subjective ultrasound findings per AAO-HNS-neck level

A							
AAO-HNS neck LN level	AUC	OCP	SNS	SPC	PPV	NPV	YID
2D Roundness index: $\frac{l}{b}$							
IA	0.90	1.50	0.89	0.91	0.44	0.99	0.80
IB	0.84	1.61	0.78	0.81	0.39	0.96	0.59
IIA	0.79	1.74	0.62	0.90	0.43	0.95	0.52
IIB	0.96	1.61	0.88	0.96	0.70	0.99	0.84
III	0.83	2.01	0.69	0.86	0.32	0.97	0.55
IV	0.88	1.47	0.80	0.94	0.62	0.97	0.74
VA	0.93	1.78	1.00	0.82	0.50	1.00	0.82
VB	0.94	1.56	1.00	0.81	0.57	1.00	0.81
All	0.83	1.79	0.74	0.82	0.34	0.96	0.56
2D Roundness index: $\frac{{\varvec{l}}}{{\varvec{d}}2}$							
IA	0.91	1.65	0.89	0.87	0.35	0.99	NA
IB	0.85	1.63	0.80	0.85	0.46	0.96	NA
IIA	0.79	1.94	0.66	0.84	0.34	0.95	NA
IIB	0.96	1.73	0.88	0.99	0.88	0.99	NA
III	0.85	2.13	0.73	0.89	0.37	0.97	NA
IV	0.86	1.72	0.70	0.86	0.39	0.96	NA
VA	0.99	1.69	1.00	0.97	0.86	1.00	NA
VB	0.91	1.45	1.00	0.88	0.67	1.00	NA
All	0.83	1.85	0.72	0.84	0.36	0.96	NA
3D Roundness index: ${\left(\frac{{\varvec{l}}}{{\varvec{b}}}\right)}^{2}{\varvec{x}}\boldsymbol{ }{\left(\frac{{\varvec{l}}}{{\varvec{d}}1}\right)}^{2}{\varvec{x}}\boldsymbol{ }{\left(\frac{{\varvec{l}}}{{\varvec{d}}2}\right)}^{2}$							
IA	0.93	6.40	0.89	0.95	0.57	0.99	0.84
IB	0.85	7.03	0.72	0.90	0.53	0.95	0.62
IIA	0.79	18.24	0.65	0.83	0.33	0.95	0.48
IIB	0.99	16.87	1.00	0.88	0.50	0.95	0.88
III	0.83	24.89	0.69	0.89	0.37	0.97	0.58
IV	0.85	31.02	1.00	0.59	0.23	1.00	0.59
VA	0.96	11.96	1.00	0.88	0.60	1.00	0.88
VB	0.92	5.22	1.00	0.88	0.67	1.00	0.88
All	0.83	14.97	0.71	0.84	0.35	0.96	0.55

B							
AAO-HNS neck LN level	AUC	OCP in mm	SNS	SPC	PPV	NPV	YID
l (length)							
IA	0.38	7.35	0.56	0.55	0.09	0.55	0.11
IB	0.72	12.95	0.39	0.21	0.07	0.21	− 0.40
IIA	0.71	14.95	0.35	0.33	0.06	0.33	− 0.32
IIB	0.91	10.70	0.00	0.29	0.00	0.29	− 0.71
III	0.68	11.65	0.30	0.38	0.04	0.38	− 0.33
IV	0.53	6.10	0.40	0.78	0.18	0.78	0.18
VA	0.58	7.55	0.67	0.77	0.36	0.77	0.44
VB	1.00	12.55	0.00	0.00	0.00	0.00	− 1.00
All	0.70	14.55	0.47	0.21	0.07	0.21	− 0.33
b (width)							
IA	0.86	4.95	0.78	0.83	0.27	0.98	0.61
IB	0.85	8.75	0.63	0.95	0.67	0.94	0.58
IIA	0.86	7.05	0.76	0.83	0.35	0.97	0.59
IIB	1.00	7.15	1.00	0.99	0.89	1.00	0.99
III	0.88	4.95	0.79	0.83	0.30	0.98	0.62
IV	0.72	5.90	0.60	0.92	0.50	0.95	0.52
VA	0.70	4.85	0.67	0.79	0.36	0.93	0.46
VB	1.00	8.05	1.00	1.00	1.00	1.00	1.00
All	0.85	7.05	0.67	0.89	0.42	0.96	0.56
d1 (diameter 1)							
IA	0.73	11.25	0.60	0.90	0.45	0.94	0.50
IB	0.79	11.25	0.61	0.88	0.44	0.93	0.49
IIA	0.79	13.05	0.63	0.84	0.32	0.95	0.47
IIB	0.94	9.45	1.00	0.77	0.33	1.00	0.77
III	0.79	8.15	0.76	0.69	0.18	0.97	0.45
IV	0.58	7.55	0.60	0.72	0.21	0.94	0.32
VA	0.55	5.65	0.50	0.82	0.33	0.90	0.32
VB	1.00	11.10	1.00	1.00	1.00	1.00	1.00
All	0.77	11.45	0.59	0.85	0.32	0.94	0.44
d2 (diameter 2)							
IA	0.89	4.80	0.78	0.84	0.28	0.98	0.62
IB	0.86	8.55	0.64	0.97	0.76	0.94	0.61
IIA	0.86	7.75	0.68	0.92	0.50	0.96	0.60
IIB	1.00	6.55	1.00	0.99	0.89	1.00	0.99
III	0.89	4.85	0.75	0.88	0.36	0.98	0.64
IV	0.73	4.65	0.70	0.80	0.30	0.96	0.50
VA	0.62	4.70	0.67	0.76	0.33	0.93	0.43
VB	1.00	8.05	1.00	1.00	1.00	1.00	1.00
All	0.86	6.45	0.69	0.87	0.40	0.96	0.56

c						
AAO-HNS neck LN level	SNS	SPC	PPV	NPV	lr	prev
IA	0.67	0.98	0.75	0.97	37.67	0.07
IB	0.83	0.99	0.92	0.97	73.21	0.14
IIA	0.73	0.99	0.92	0.97	94.79	0.11
IIB	1.00	1.00	1.00	1.00	Inf	0.10
III	0.77	0.99	0.86	0.98	65.52	0.08
IV	0.70	1.00	1.00	0.96	Inf	0.11
VA	1.00	1.00	1.00	1.00	Inf	0.15
VB	1.00	1.00	1.00	1.00	Inf	0.20
All	0.79	0.99	0.91	0.97	81.85	0.11

(a) Values for the area under the curve (AUC), optimal cutoff point (OCP), sensitivity (SNS), specificity (SPC), positive predictive value (PPV), negative predictive value (NPV), and Youden index (YID) for the 2D- and 3D-roundness indices (see Fig. 1 and Materials and Methods under “US Examination of Neck LNs”) according to neck LN level and sublevel per the AAO-AHNS classification. (b) Values for the area under the curve (AUC), optimal cutoff point (OCP), sensitivity (SNS), specificity (SPC), positive predictive value (PPV), negative predictive value (NPV), and Youden index (YID) for the individual distances l, b, d1, and d2 (see Fig. 1 and Materials and Methods under “US Examination of Neck LNs”) according to neck LN level and sublevel per the AAO-AHNS classification. (c) Performance of the subjective ultrasound findings in detecting LN metastases (including the likelihood ratio (lr) and prevalence (prev) according to neck LN level and sublevel per the AAO-AHNS classification

### The predictive performance of US increases with metastasis size

To determine the performance of the objective criteria and the subjective ultrasound findings as a function of the size of the metastasis, we assessed metastasis size by measuring the diameters of the tumor deposits inside the LNs in two dimensions microscopically, and the longest diameter was used for evaluation. Because of the otherwise insufficient number of LNs, the same OCPs (the respective ideal values for all levels (I–VI), Table 1, under “all”), were jointly used for all LNs from the different neck LN levels except for subjective ultrasound findings. Metastases were categorized based on the UICC classification as follows: isolated tumor cells (ITCs) and micrometastases had a longest diameter ≤ 2 mm (Sobin et al. 2011), and macrometastases, with a longest diameter > 2 mm, were grouped in 2-mm increments up to a maximum diameter of 10 mm, and as metastases with a longest diameter > 10 mm. Among all criteria (indices and subjective US findings), the predictive performance increased (linearly) with increasing size of the metastases (Table 2). The 3D roundness index was not superior to the 2D index. Among the individual distances, d2 showed the best performance with a cutoff point of 6.45 mm (Table 2). The data also showed that subjective assessment is insufficient for the detection of micrometastases, ITCs and macrometastases up to 8 mm.

**Table 2:** Performance of the two roundness indices (2D and 3D); the individual measured distances l, b, d1, and d2; and the subjective ultrasound findings separated according to the greatest extent of the metastases within the LNs of less than 2 mm (interstitial tumor cells (ITCs) and micrometastases) in 2-mm increments to more than 10 mm in the longest diameter

Maximum diameter of metastasis	≤ 2 mm (ITCs or micrometastases)	> 2 and ≤ 4 mm	> 4 and ≤ 6 mm	> 6 and ≤ 8 mm	> 8 and ≤ 10 mm	> 10 mm
2D—roundness ($\frac{{\varvec{l}}}{{\varvec{b}}}$ < 1.79)						
n pos	8	20	33	18	28	107
n	23	39	44	30	35	121
SNS	0.35	0.51	0.75	0.60	0.80	0.88
3D—roundness (${\left(\frac{l}{b}\right)}^{2}x {\left(\frac{l}{d1}\right)}^{2}x {\left(\frac{l}{d2}\right)}^{2}$ < 14.97)						
n pos	9	17	34	18	27	104
n	23	39	44	30	35	121
SNS	0.39	0.44	0.77	0.60	0.77	0.86
l (> 14.55 mm)						
n pos	9	18	10	7	15	99
n	23	39	44	30	35	121
SNS	0.39	0.46	0.23	0.23	0.43	0.82
b (> 7.05 mm)						
n pos	9	16	20	15	26	110
n	23	39	44	30	35	121
SNS	0.39	0.41	0.46	0.50	0.74	0.91
d1 (> 11.45 mm)						
n pos	7	14	12	9	22	110
n	23	39	44	30	35	121
SNS	0.30	0.36	0.27	0.30	0.63	0.91
d2 (> 6.45 mm)						
n pos	11	18	20	15	28	112
n	23	39	44	30	35	121
SNS	0.48	0.46	0.46	0.50	0.80	0.93
Subjective ultrasound findings						
n pos	8	18	29	20	33	121
n	23	39	44	30	35	121
SNS	0.35	0.46	0.66	0.67	0.94	1.00
Graph 2b: Performance of the indices and individual diameters depending on the metastasis size within the LNs						


Due to the otherwise insufficient number of LNs per neck region, the optimal cutoff points for all regions (Table 1) were used. (a) Tabular representation of the data: The total number of positive results per LN and the resulting sensitivity are given. As expected, shorter diameters (b, d2) show better performance than longer diameters (l, d1)

Regarding the objective criteria, namely, 2D, 3D, l, b, d1, and d2, the cutoff values over all neck levels were assumed in each case, thus tending to result in poorer performance than with exact separation by individual neck levels, as in the subjective findings. *n pos.* number of positive LNs, *n* number of LNs, *SNS* sensitivity; Graph 2b: Graphical processing of the data

### Comparison of clinical and pathological N status

Beyond analyses based on single LNs, we wanted to determine the contribution of sonography to N-staging of the patients. To do this, each patient was assigned a cN status on the basis of measurements of the individual distances l, b, d1, and d2 (with respective cutoffs) and subjective US findings, and the predictive performance of the cN status was assessed by examining the pathological N status (pN status; Table 3).

**Table 3:** Performance of the two roundness indices (2D and 3D), the individual measured distances l, b, d1, and d2 and the subjective ultrasound findings in the prediction of the pathological N status (pN status)

Clinical N status based solely on 2D—roundness ($\frac{{\varvec{l}}}{{\varvec{b}}}$) vs. pathological N status							
	cN0	cN1	cN2	cN2a	cN2b	cN2c	cN3
pN0	51	34			16	31	1
pN1	10	12			7	13	
pN2			1				
pN2a	1			2			
pN2b	1	6			27	11	
pN2c	2	1			3	13	
pN3						1	4

Clinical N status based solely on 3D—roundness (${\left(\frac{l}{b}\right)}^{2}x {\left(\frac{l}{d1}\right)}^{2}x {\left(\frac{l}{d2}\right)}^{2}$) vs. pathological N status							
	cN0	cN1	cN2	cN2a	cN2b	cN2c	cN3
pN0	61	24			13	35	
pN1	6	15			11	11	
pN2			1				
pN2a	1			2			
pN2b	3	5			27	9	
pN2c	1				4	14	
pN3						2	3

Clinical N status based solely on l (length) vs. pathological N status							
	cN0	cN1	cN2	cN2a	cN2b	cN2c	cN3
pN0	13	12			41	67	
pN1	1	12			10	17	
pN2			2				
pN2a					2	1	
pN2b	2	3			19	21	
pN2c						20	
pN3						2	3

Clinical N status based solely on b (width) vs. pathological N status							
	cN0	cN1	cN2	cN2a	cN2b	cN2c	cN3
pN0	52	27			16	38	
pN1	8	18			10	5	
pN2			2				
pN2a					2	1	
pN2b	2	7			23	13	
pN2c		1			2	16	
pN3							5

Clinical N status based solely on d1 (diameter 1) vs. pathological N status							
	cN0	cN1	cN2	cN2a	cN2b	cN2c	cN3
pN0	32	32			27	42	
pN1	8	14			11	9	
pN2			2				
pN2a				1	2		
pN2b	5	5			22	13	
pN2c		1				17	
pN3						2	3

Clinical N status based solely on d2 (diameter 2) vs. pathological N status							
	cN0	cN1	cN2	cN2a	cN2b	cN2c	cN3
pN0	77	25			9	22	
pN1	11	16			6	8	
pN2			2				
pN2a				2	1		
pN2b	2	8			30	4	
pN2c	1	1			2	16	
pN3							5

Clinical N status based solely on subjective ultrasound findings vs. pathological N status							
	cN0	cN1	cN2	cN2a	cN2b	cN2c	cN3
pN0	128	2			1	2	
pN1	17	22			2		
pN2			2				
pN2a				3			
pN2b	2	8		1	34		
pN2c	4	1			2	12	
pN3						2	3

Clinical N status (N0, N +) based solely on 2D—Roundness ($\frac{{\varvec{l}}}{{\varvec{b}}}$) vs. pathological N status (N0, N +)		
	cN0	cN +
pN0	51	82
pN +	14	101

Clinical N status (N0, N +) based solely on 3D—Roundness (${\left(\frac{l}{b}\right)}^{2}x {\left(\frac{l}{d1}\right)}^{2}x {\left(\frac{l}{d2}\right)}^{2}$) vs. pathological N status (N0, N +)		
	cN0	cN +
pN0	61	72
pN +	11	104

Clinical N status (N0, N +) based solely on l (length) vs. pathological N status (N0, N +)		
	cN0	cN +
pN0	13	120
pN +	3	112

Clinical N status (N0, N +) based solely on b (width) vs. pathological N status (N0, N +)		
	cN0	cN +
pN0	52	81
pN +	10	105

Clinical N status (N0, N +) based solely on d1 (diameter 1) vs. pathological N status (N0, N +)		
	cN0	cN +
pN0	32	101
pN +	13	102

Clinical N status (N0, N +) based solely on d2 (diameter 2) vs. pathological N status (N0, N +)		
	cN0	cN +
pN0	77	56
pN +	14	101

Clinical N status (N0, N +) based solely on subjective ultrasound findings vs. pathological N status (N0, N +)		
	cN0	cN +
pN0	128	5
pN +	23	92

This was based on the respective optimal cutoff points per (sub)region (IA–VB). For each parameter and for the subjective ultrasound findings, only the individual values per LN were used, and a clinical N status (cN status) was established and correlated with the final pathological N status (pN status). Concordant results are found on the diagonal; to the left of the diagonal are underestimated findings, and to the right of the diagonal are overestimated findings. The numbers indicate the frequency of treatment occasions (248 in total) per respective cN/pN constellation. The left table separates according to the individual N stages, and the right-hand part of the table differentiates only between N0 and N + patients

In the present study, the clinical relevance of pathological upstaging, specifically from cN0 to cN+, merits particular attention. As delineated in Table 3a, among the cohort of 151 patients initially designated as cN0 based on ultrasonographic evaluation, 128 were histopathologically confirmed as pN0. Conversely, 17 patients were upstaged to pN1, 2 to pN2b, and 4 to pN2c. A comprehensive analysis of the LNs accountable for the diagnostic discordance is elucidated in Suppl. Table 1. The ultrasonographic dimensions of the LNs ranged from 8 to 29 mm (mean = 16.01 mm, median = 15.7 mm), whereas the histological dimensions varied from 4.5 to 20 mm (mean = 10.6 mm, median = 10 mm). Furthermore, the dimensions of the metastatic foci within the LNs spanned 0.3 to 8 mm (mean = 3.67 mm, median = 3.2 mm) (Suppl. Table 1). All deviations of the cN values from the pN values were first included unweighted in Cohen’s κ calculation. Regarding the roundness indices, the 3D index (κ = 0.330) was superior to the 2D index (κ = 0.271). Among the individual distances, d2 with κ = 0.438 remained the best at correctly predicting pN status, whereas l was the worst. Overall, subjective US findings performed best, at κ = 0.713. Because any weighting (e.g., weighted Cohen’s κ (Cohen 1968)) would have led to enhancement of errors, we did not calculate a weighted Cohen’s κ because the performance was already too poor to be reliable. However, the performance improved somewhat by allowing only a binary decision, namely, cN+ vs. cN0. Again, the 3D roundness index (κ = 0.3504) was superior to the 2D roundness index (κ = 0.2517), and among single distances, d2 (κ = 0.4462) was best for making the N+ vs. N0 decision (Table 4). As before, the subjective ultrasound findings were best, with κ = 0.7706, but they were still below the limit of “almost perfect” (κ = 0.8) (Watson and Petrie 2010). In 4 cases, cN status was correctly anticipated despite the wrong LN being categorized as being suspected of containing a metastasis.

**Table 4:** Cohen’s kappa index for the prediction of pN status through the 2D-roundness index l/b and 3D-roundness index ${\left(\frac{l}{b}\right)}^{2}x {\left(\frac{l}{d1}\right)}^{2}x {\left(\frac{l}{d2}\right)}^{2}$ as well as through the individually measured distances l, b, d1, and d2

Distance resp. index	Cohen’s κ index for individual N stages			Performance in terms of discrimination pN0 vs. pN +							
	Cohen’s κ	Lower CI	Upper CI	Cohen’s κ	Accuracy	Lower CI	Upper CI	SNS	SPC	PPV	NPV
2D-roundness	0.270	0.189	0.351	0.2517	0.6129	0.5510	0.6714	0.8783	0.3835	0.5519	0.7846
3D-roundness	0.330	0.247	0.412	0.3504	0.6653	0.6045	0.7211	0.9043	0.4586	0.5909	0.8472
l	0.154	0.089	0.219	0.0670	0.5040	0.4422	0.5667	0.9739	0.0977	0.4828	0.8125
b	0.308	0.227	0.388	0.2918	0.6331	0.5715	0.6906	0.9130	0.3910	0.5645	0.8387
d1	0.206	0.131	0.281	0.1213	0.5403	0.4782	0.6013	0.8870	0.2406	0.5025	0.7111
d2	0.439	0.354	0.524	0.4462	0.7177	0.6587	0.7701	0.8783	0.5789	0.6433	0.8462
Subjective US findings	0.713	0.636	0.790	0.7706	0.8871	0.8417	0.9207	0.8000	0.9624	0.9485	0.8477

Left three columns: Cohen’s kappa index. A commonly used judgment rule for κ is given in the following classification (Watson and Petrie 2010): poor, < 0.0 (upper limit); slight, 0.0–0.2; fair, > 0.2–0.4; moderate, > 0.4–0.6; substantial, > 0.6–0.8; and almost perfect; > 0.8–1.0 (upper limit). Right seven columns: accuracy, lower confidence interval (CI), upper CI, sensitivity (SNS), specificity (SPC), positive predictive value (PPV) and negative predictive value (NPV) for roughly predicting a positive LN status (N +) versus a negative (N0) LN status

In summary, we evaluated objective and subjective morphologic US criteria to predict metastases in neck LNs. They show different values depending on the neck level and their performance in micrometastases is insufficient, but improves with increasing size of metastases.

## Discussion

This work was undertaken to document the current performance of US in the noninvasive detection of carcinoma metastases in neck LNs. Particular emphasis was placed on objective morphological criteria (especially length measurements and roundness indices) because of the common prejudice that US is a subjective method (Wolff 2019).

The study involved an analysis of concordance between two datasets: sonographically and histopathologically examined LNs. Our results showed that the two datasets had high congruence in terms of the N stage distribution based on subjective US findings, even though the number of histopathologically examined LNs was > 1.6 × higher than that of sonographically examined LNs (7237 vs. 4539). The number of metastases detected was also > 1.6 × higher among specimens examined histopathologically, with 443 histopathologically examined and 265 US-examined specimens having metastases. As expected, more LNs with metastases were found overall in the histopathological workup because the workup involved microscopy. Additionally, the proportion of LNs with metastases by microscopy (6.17%) was somewhat higher than that by sonography (5.82%). However, this may be because selective ND usually focuses on the side with the primary tumor and is often limited to levels most likely to be affected by metastases, depending on the location of the primary tumor; conversely, when US is performed, all levels are examined equally (Wolff 2019). Nevertheless, in our study, single ITCs and micrometastases could not be detected with sufficient reliability by US. The best performance for metastases between 2 and 6 mm was evident with the 2D and 3D roundness indices; however, for metastases larger than 6 mm, subjective US had the highest accuracy.

The cutoff values for objective parameters (distances and indices) displayed a considerable spread depending on the AAO-HNS (sub)neck LN level, consistent with a previous report that did not have an accompanying histological assignment (Ying et al. 1999). Thus, consideration of LNs separately according to neck level improves the performance of individual parameters. Here, valid cutoff values were determined for all parameters, with some deviation from previously reported values. For example, the cutoff value for the 2D roundness index across all levels was 1.79 instead of the previously assumed value of 2 (Solbiati et al. 1992). Additionally, compared to the 2D index, the 3D index showed slightly better specificity (84% vs. 82%) but lower sensitivity (71% vs. 74%). As its application is very cumbersome due to the large spread of values across levels, the 3D roundness index does not constitute a diagnostic gain. In the case of a metastasis originating from one or a few cells and increasing in size concentrically in spherical form, the smaller the diameter is, the earlier the metastasis must "strike out". In this study, the order of distances sorted according to how fast they change in response to a metastasis, was d2 > b > d1 > l. Hence, the diameter d2 is the best single distance to predict metastasis. The cutoff value was 6.45 mm across all levels, with values ranging from 4.8 to 8.55 mm depending on the neck level. Previous reports have used 8 mm for the diameter corresponding to our b (Bruneton et al. 1984) and 11 mm for the minimum axial diameter (corresponding to our d2) for LNs at level II (van den Brekel et al. 1998) and 10 mm (Close et al. 1989; Kelly and Curtin 2017) or 12 mm (Sun et al. 2015a, b) for LNs at the remaining levels (I, III–VI). Thus, by direct comparison of sonography and histology of a large number of individual LNs, new, robust cutoff values for roundness indices and individual distances could be determined.

A comparable work from 2019, also comparing US and histology results of the same LN, detected 67 LNs from 34 patients with surgically treated HNSCC across all levels. The cutoff values in that study were 1.2 for the 2D roundness index, 13.6 mm for l, 14 mm for b, and 9.4 mm for “thickness”, which in their work corresponded to the dimension of the LN that is perpendicular to the body surface and in our work most closely equates to d2 (Nishio et al. 2019). The disadvantage of individual parameters continues to be their low specificity. For example, cortical hyperplasia or localized follicular hyperplasia can also lead to focal enlargement of an LN (Kelly and Curtin 2017). So, although the individual distances and indices may well approach subjective findings in sensitivity for metastases from 6 mm on, specificity remains their major weakness.

Regarding other imaging methods, although they are not the focus of our study, it is worth noting that the corresponding performance of CT and MRI for the prediction of LN metastasis has been investigated. A recent meta-analysis of 63 studies and 3029 patients (Sun et al. 2015a, b) revealed that, assuming a single LN as a unit of comparison, CT has a sensitivity of 0.77 and a specificity of 0.85, and the corresponding values for MRI are 0.72 and 0.84, respectively. However, among the studies examined, none reported such a meticulous 1:1 node-by-node comparison as that performed in our current study. CT examination combined with FDG-PET has been shown to be the most successful method for LN staging in several meta-analyses (Yongkui et al. 2013; Sun et al. 2015a, b). In a recent meta-analysis of 24 studies with 1,270 patients, the sensitivity and specificity values for ^18^F-FDG-PET/CT were 0.91 and 0.87 per patient, 0.84 and 0.83 per neck side and 0.80 and 0.96 per neck level (Sun et al. 2015a, b). Within another otherwise promising meta-analysis of 24 studies that included a previous study from our own group (Heusch et al. 2014), the sensitivity and specificity values we obtained with meticulous 1:1 node-by-node mapping and comparison analogous to the present work were much less favorable (31.0 and 97.3, respectively), due mainly to small LNs that were not detected at all or not detected as positive (Sun et al. 2015a, b). In summary, the published data from the cross-sectional imaging methods show high sensitivities in some cases, but lag behind our US data in specificity.

As expected, the subjective US findings had the best performance, and their strength is their specificity. Their greatest weakness was in the detection of small metastatic deposits; specifically, metastases less than 8 mm in diameter were not detected with adequate confidence. The subjective US findings also showed strong variations in performance depending on the neck level. In particular, it is worth mentioning level IA, with its small, rather roundish LNs, and IIA, where the LNs are located deeper in the neck and hidden within the complex anatomy, making them subjectively more difficult to examine than LNs at a more superficial level, such as IB or IV. Unfortunately, IA and IIA are the clinically more important neck levels (Wolff 2019) The lack of specificity of single diameters and the low sensitivity are also reflected in the decision on cN stage based only on the US modality: only the binary decision of cN0 vs. cN+ based on subjective findings was valid enough to have clinical relevance in our study. Moreover, computer-assisted risk analysis using retrospective data showed that in the case of oral cavity carcinoma, elective ND is clearly indicated if the probability of occult metastasis is greater than 20% (Weiss et al. 1994). This would, therefore, require a NPV of more than 80% (Nishio et al. 2019), which was achieved in our study with small distances (b, d1, and d2), the two indices (2D and 3D), and subjective sonographic findings.

The goal of any imaging procedure must be to compete with the gold standard, namely, histopathologic findings, to obviate the need for surgical staging in the future. In addition to avoiding surgical risks (Gane et al. 2017), preservation of LNs is important because tumor-draining LNs (TDLNs) have been proven to be necessary to overcome immune checkpoint inhibitor blockade resistance in recent years, and LN preservation is becoming increasingly important in the adjuvant and neoadjuvant setting in HNSCC (van Pul et al. 2021). TDLNs, eventually supported by intra- or peritumoral injection of immune checkpoint inhibitors (CTLA-4- or PD-1 blocking antibodies) (Fransen et al. 2013) as a site of antigen exchange between different DC subsets, play a central role in the induction of effective systemic antitumor T cell immunity (Borst et al. 2018), and their surgical removal impairs clinical outcomes (Chamoto et al. 2017). However, our meticulous preparation of samples with extensive node-by-node assignment is suitable for defining the gap to histopathological examination. Moreover, there remains a clear lack of sensitivity with regard to the detection of small deposits. To ameliorate this, the implementation of superior high-resolution B-scans is recommended. Moreover, the inclusion of microvascular flow imaging (MVFI) could facilitate the visualization of intricate changes in hilar and peripheral blood vessels within the LNs. These vessels often undergo early pathological alterations, manifesting as split vessels, vessel tortuosity, and vessel breaks (Ahuja and Ying 2005; Aziz et al. 2022). The adoption of shear-wave elastography (SWE) could serve as an additional method for enhancing diagnostic accuracy (Lerchbaumer et al. 2022).

There are certainly imitations of the current study: above all, the large amount of time required for ultrasound examination and sample preparation in the operating room, which made a multicenter setting difficult. This was the reason for the long time needed for data acquisition. Another significant limitation of this study is the reliance on a single, albeit highly experienced, examiner to conduct all US evaluations. It is important to note that sonographic imaging is a skill highly dependent on the operator’s experience and expertise. The examiner in this study has substantial experience in the field, which could influence the quality and consistency of the LN measurements. Therefore, the results may not be directly generalizable to other departments or less experienced examiners.

## Conclusion

Based on our data with the meticulous 1:1 node-by-node comparison, US is currently the best documented imaging method for the detection of cervical LN metastases. As expected, subjective findings approximated the histopathologic diagnosis most closely. However, objective criteria including roundness indices and the mere measurement of individual distances also showed a high sensitivity, but a lower specificity than the subjective US findings. Its weaknesses, however, were still evident in our study, especially in the detection of small tumor deposits, and the performance of US is currently not sufficient to replace histopathological assessment. Furthermore, it’s crucial to note that the results are highly influenced by the expertise of the examiner involved. Sonographic imaging is inherently operator-dependent, making the experience of the investigator a critical factor for obtaining reliable and consistent measurements. The integration of high-frequency b-mode ultrasound, microvascular flow imaging and shear-wave elastography as multiparametric ultrasound holds the promise to eventually close the small gap to histopathological workup and to make surgical staging unnecessary one day. However, the results presented warrant another prospective study of the wait & watch policy in cN0-HNSCC patients.

### Supplementary Information

Below is the link to the electronic supplementary material.

Suppl. file1 (DOCX 41 KB)

Suppl. Figure 1: Representative analysis of sonographically detected and surgically excised lymph nodes (LNs) during neck dissection (ND) in patient #170. A: Map of the AAO-AHNS-based neck levels (indicated by 1A through 6). and B. The schematically drawn LNs observed by US examination on the day before surgery. The LNs were not labeled further, meaning that they were classified as tumor free. The first, second, third and fourth numbers indicate the lengths of the distances l x b x d1 x d2 in millimeters. The circled numbers correspond to the numbers on the pathology containers and on the submission form for the histopathological assessment. LNs were identified individually during ND, correlated with US findings and placed into individual containers for individual histopathological assessment per single LN (PDF 118 KB)

Suppl. file3 (PDF 1101 KB)

Suppl. Figure 2: Photograph of the prepared LNs packed in formalin in individually labeled transport containers for individual examination at the Institute of Pathology (PPTX 901 KB)

Suppl. Figure 3: CRF of the individually designed "Lymphsono" database based on the Omnicomm software (PDF 17 KB)

Suppl. Figure 4: Representative false positive results. A. a: LN #1 of a 28-year-old female patient is shown. She reported a steadily increasing painless swelling for 2 weeks prior to the examination. b: Sonography showed two rounded LNs with a chaotic hypointense internal echo and clear pathological vascularization. The LN was classified as bearing a metastasis. The two indices (2D: 1.42, OCP for level IIA: 1.74 and 3D: 2.83, OCP for level IIA: 18.24) were also false positive. Histopathology revealed a LN with low-grade, chronic, unspecific lymphadenitis and salivary gland tissue heterotopia as well as adjacent other serous salivary gland parenchyma with cystic duct dilatation and no evidence of malignancy. c-e: The dilated gland lumina are indicated by asterisks. e: Gland acini and small ducts are indicated by black lines. Scale bars are presented in the lower left corners of the images, and the magnifications are as follows: 3 mm, 0.9x (c); 400 µm, 6.5x (d); and 90 µm, 24.4x (e). B. a: The level IIA lymph node shown was categorized as “probably filia” (counted as positive) in the subjective findings due to the spatial proximity to a metastasis in the bifurcation area and an eccentric cortical hypertrophy (red circles in b). The two indices (2D: 2.39, OCP for level IIA: 1.74 and 3D: 36.66, OCP for level IIA: 18.24) were correctly negative. c-e: Histopathology revealed a nonmetastatic lymph node with secondary follicles, and the tissue was examined at increasing magnifications. Representative germinal centers are indicated by asterisks. Scale bars are presented in the lower left corners of the images, and the magnifications are as follows: 2 mm, 1.3x (c); 300 µm, 7.8x (d); and 200 µm, 20x (e). C. a: The level III/IV LN shown was rated as suspicious in the subjective findings due to its spatial proximity to a metastasis in the bifurcation area and eccentric cortical hypertrophy (red circles in b). The LN was rated as negative for the evaluation. The two indices (2D: 1.63, OCP for level III, 2.01, and 3D index: 35.91, OCP for level III, 24.89) were false positive and correctly negative, respectively, upon the results of histopathology. c-e: A large lymph node with paracortical and secondary lymphofollicular hyperplasia (representative germinal centers indicated by asterisks) is shown, and no metastasis was observed histologically. Scale bars are presented in the lower left corners of the images, and the magnifications are as follows: 3 mm, 0.8x (c); 400 µm, 5.6x (d); and 200 µm, 20x (e) (PPTX 12834 KB)

Suppl. file7 (PPTX 14212 KB)

Suppl. file8 (PPTX 11298 KB)

Suppl. Figure 5: Representative false negative results: A. LN #3 of patient #136 was subjectively incorrectly classified as tumor free. Both the 2D roundness index of 1.69 (OCP for level IB: 1.61) and the 3D index of 8.18 (OCP for level IB: 7.03) incorrectly indicated "no metastasis". A metastasis of 6 x 4 mm in maximum diameter was found by histopathology. a: In the LN map, LN #3 is indicated by a red circle. b: B-scan of the LN in two perpendicular planes. c-e: Increasing magnifications of the LN metastasis with central necrosis of a squamous cell carcinoma. The borders of the metastasis are indicated by the black line, scale bars are presented in the lower left corners of the images, and the magnifications are as follows: 2 mm, 1.8x (c); 500 µm, 4.8x (d); and 100 µm, 21.2x (e). B. LN #18 of patient #132 was classified as metastasis-affected in the subjective ultrasound findings, as a small tumor with a maximum diameter of 7 mm was detected within the LN. Both the 2D index (2.94, OCP for level III: 2.01) and the 3D index (403.93, OCP for level III: 24.89) results were false negatives. The metastasis suspected in the B-scan was confirmed histopathologically (3 mm in maximum diameter). a: In the LN map, LN #18 is indicated by a red circle, b and c: B-scan of the LN in two perpendicular planes. The LN was measuring 18.8 x 6.4 x 8.2 x 6.3 mm and the metastasis within it 6.9 x 5.8 x 6.1 x 4.9 mm. d-f: Increasing magnifications of the LN containing a metastasis of nonkeratinizing squamous cell cancer, with increasing magnifications. The borders of metastasis are indicated by the black line, and scale bars are presented in the lower left corners of the images. The magnifications are as follows: 2 mm, 1.7x (d); 400 µm, 5.3x (e); and 90 µm, 23.2x (f). C. For LN #7 of patient #44, a small marginal finding of approximately 2 mm was detected in the B-scan, and this was not classified as a metastasis because of the hyperintense internal echo. The two indices (2D: 2.92, OCP in level III: 2.01 and 3D: 3670.17, OCP in level III: 24.89) incorrectly indicated a "tumor-free" status. Histopathology revealed a marginal metastasis of 2.5 mm in maximum diameter. a: In the LN map, LN #7 is indicated by a red circle. b: B-scan of the LN in two perpendicular planes. c-e: LN metastasis of keratinizing squamous cell cancer shown at increasing magnifications. The borders of the metastasis are indicated by the black line, and scale bars are presented in the lower left corners of the images. The magnifications are as follows: 2 mm, 1.4x (c); 500 µm, 4.7x (d); and 100 µm, 20.8x (e). (PPTX 13534 KB)

Suppl. file10 (PPTX 14760 KB)

Suppl. file11 (PPTX 13050 KB)

Suppl. Table 1: Excerpt from the primary database for an overview of the individual lymph nodes seen as false negatives in the subjective US examination that were responsible for upstaging from cN0 to cN+ (PDF 24 KB)

## Data Availability

The datasets generated during and analyzed during the current study are available from the corresponding author upon reasonable request.
