# Supplementary material for: Comparative analysis of diagnostic ultrasound and histopathology for detecting cervical lymph node metastases in head and neck cancer
Source: J Cancer Res Clin Oncol. 2023 Oct 12;149(19):17319–33. doi: 10.1007/s00432-023-05439-x (PMC10657327; doi:10.1007/s00432-023-05439-x)
Supplement: Supplementary file 1 — Suppl. file1 (DOCX 41 KB) [file 432_2023_5439_MOESM1_ESM.docx]

**Materials and Methods**

**US Examination of Neck LNs**

Specifically, data on external (size, shape, dorsal sound amplification, delimitation, regional distribution and eventual extracapsular extension of intranodal masses) and internal (recognizability of the hilum, eccentric cortical hypertrophy, and the presence of cystic or coagulated necrosis zones) characteristics were collected for all specimens. Vascular supply patterns were examined using color and power Doppler. In this analysis, hilar vessels, longitudinal vessels, centrally branching vessels or central vascular segments were considered indicators of reactive LNs and vascular displacement, and an aberrant vascular course, avascular foci or subcapsular vessels were considered evidence of metastatic involvement of the LNs.^1,2^ A metastasis itself usually appears as a spherical, often echo-poor, avascular, relatively sharply circumscribed mass, often with a subcortical location.

**Patient Treatment**

Patients were treated according to the German (AWMF) S3 guideline for “oral cavity carcinoma” ^3^ and the NCCN guideline for “head and neck cancers” ^4^, as amended at the time. Surgical therapy comprised (i) excision of the primary tumor and, (ii) in patients with clinically negative LN status (cN0), elective ND, mostly ipsi ND (I-IV), regardless of T category. If there was clinical suspicion of LN involvement (cN+), comprehensive ND was carried out, usually as ipsi ND (I-V). Contra ND (I-IV) was performed if (i) tumors were located in the floor of the mouth (C04) or tongue (C02); (ii) tumors were located closer than 9 mm to the midline in the lips (C00), alveolus and gingiva (C03, C04) or palate (C05); (iii) ipsilateral metastasis was suspected in staging examinations (cN+); and (iii) immediate reconstruction of the defects by primary wound closure, regional flaps or free tissue transfer, respectively, was performed. Adjuvant therapy was recommended upon postoperative reassessment according to the pTNM status and overall condition of the patient. Salvage surgery was carried out for all patients with resectable locoregional relapse following previous radiation therapy or surgery after consultation with the multidisciplinary tumor board.
