# Supplementary figures and images for: Comparative analysis of diagnostic ultrasound and histopathology for detecting cervical lymph node metastases in head and neck cancer
Source: J Cancer Res Clin Oncol. 2023 Oct 12;149(19):17319–33. doi: 10.1007/s00432-023-05439-x (PMC10657327; doi:10.1007/s00432-023-05439-x)

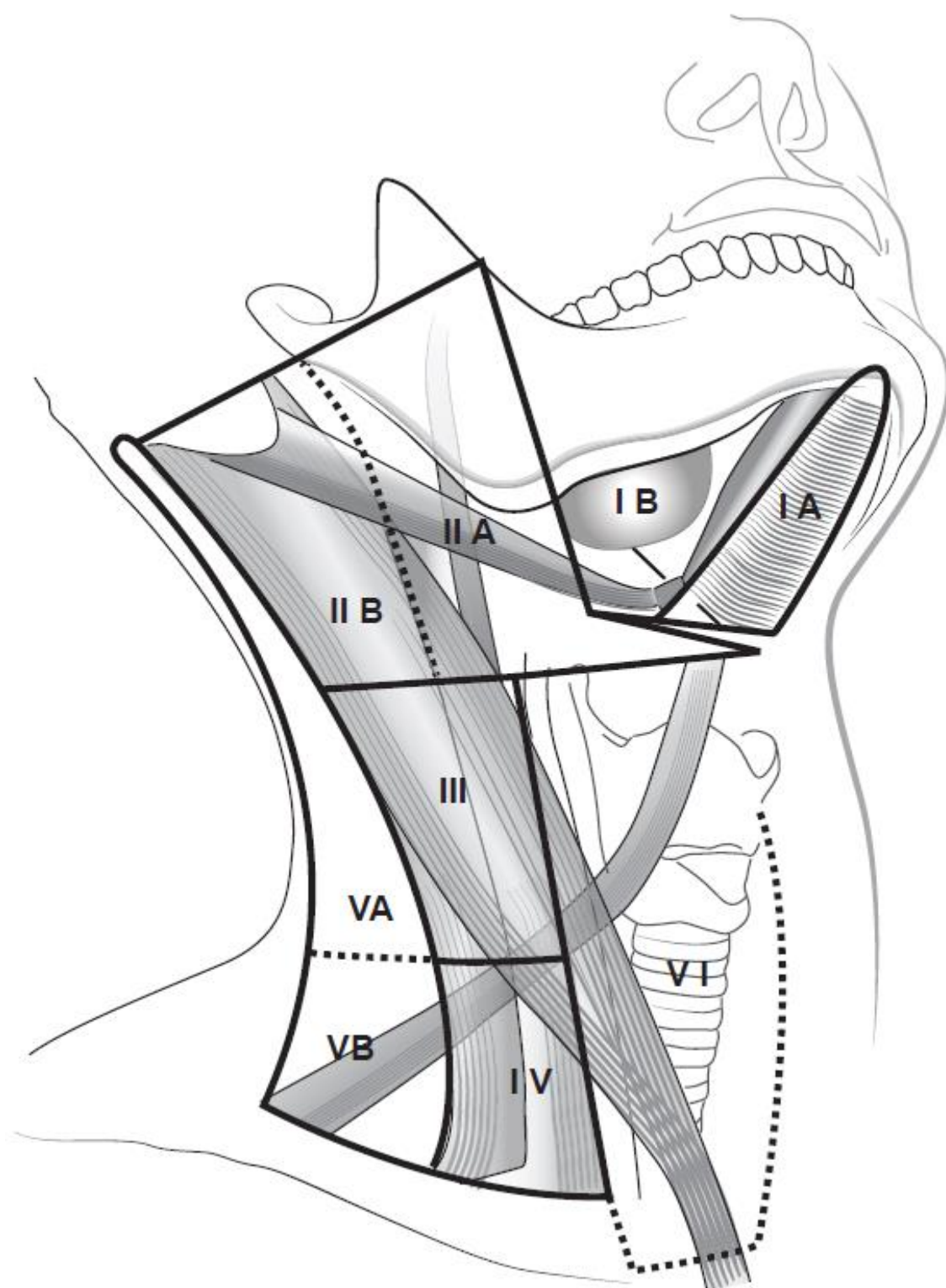

Supplement: Supplementary file 2 — Suppl. Figure 1: Representative analysis of sonographically detected and surgically excised lymph nodes (LNs) during neck dissection (ND) in patient #170. A: Map of the AAO-AHNS-based neck levels (indicated by 1A through 6). and B. The schematically drawn LNs observed by US examination on the day before surgery. The LNs were not labeled further, meaning that they were classified as tumor free. The first, second, third and fourth numbers indicate the lengths of the distances l x b x d1 x d2 in millimeters. The circled numbers correspond to the numbers on the pathology containers and on the submission form for the histopathological assessment. LNs were identified individually during ND, correlated with US findings and placed into individual containers for individual histopathological assessment per single LN (PDF 118 KB) [file 432_2023_5439_MOESM2_ESM.pdf]

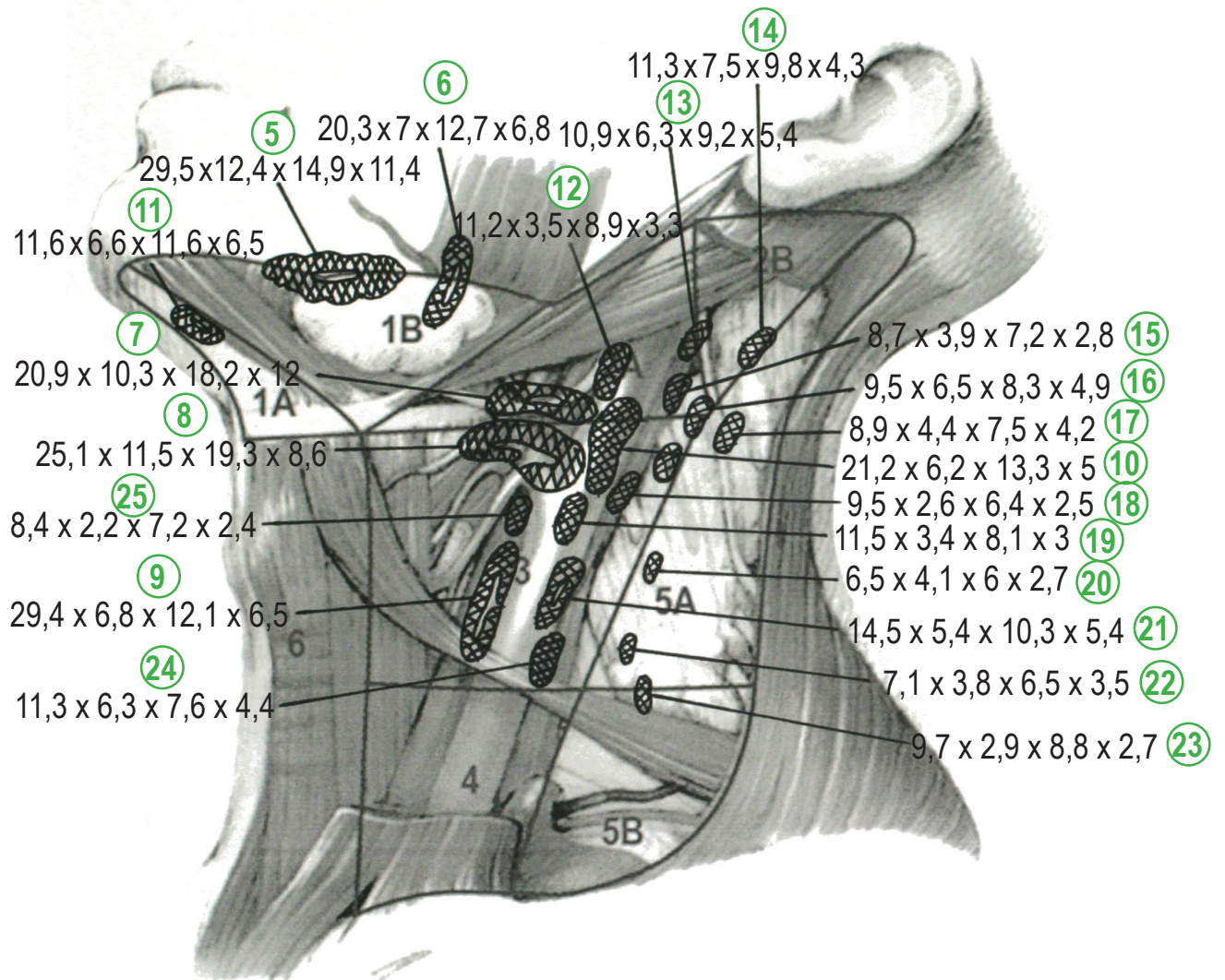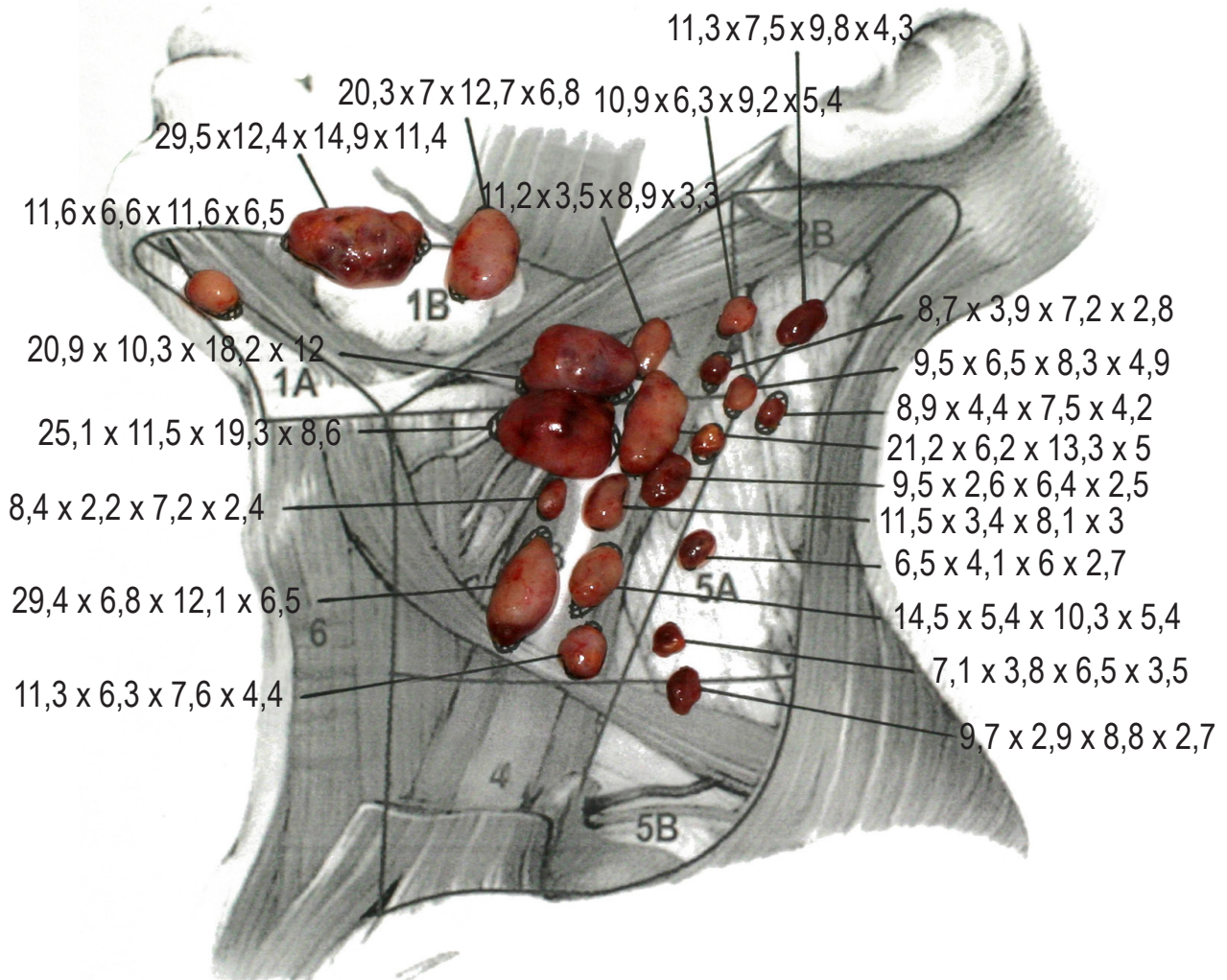

Supplement: Supplementary file 3 — Suppl. file3 (PDF 1101 KB) [file 432_2023_5439_MOESM3_ESM.pdf]

## Slide 1
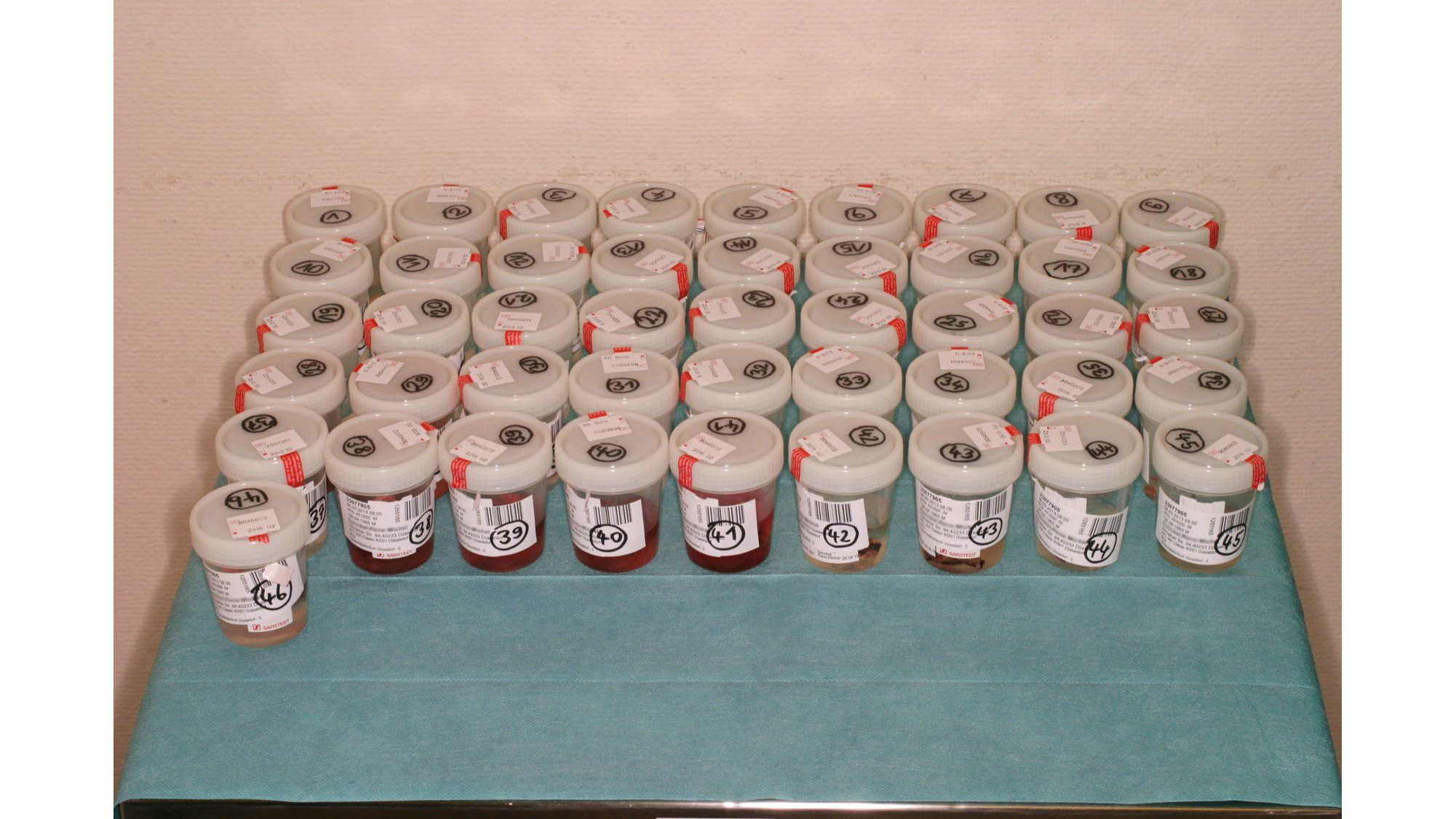

Supplement: Supplementary file 4 — Suppl. Figure 2: Photograph of the prepared LNs packed in formalin in individually labeled transport containers for individual examination at the Institute of Pathology (PPTX 901 KB) [file 432_2023_5439_MOESM4_ESM.pptx]

## Slide 1
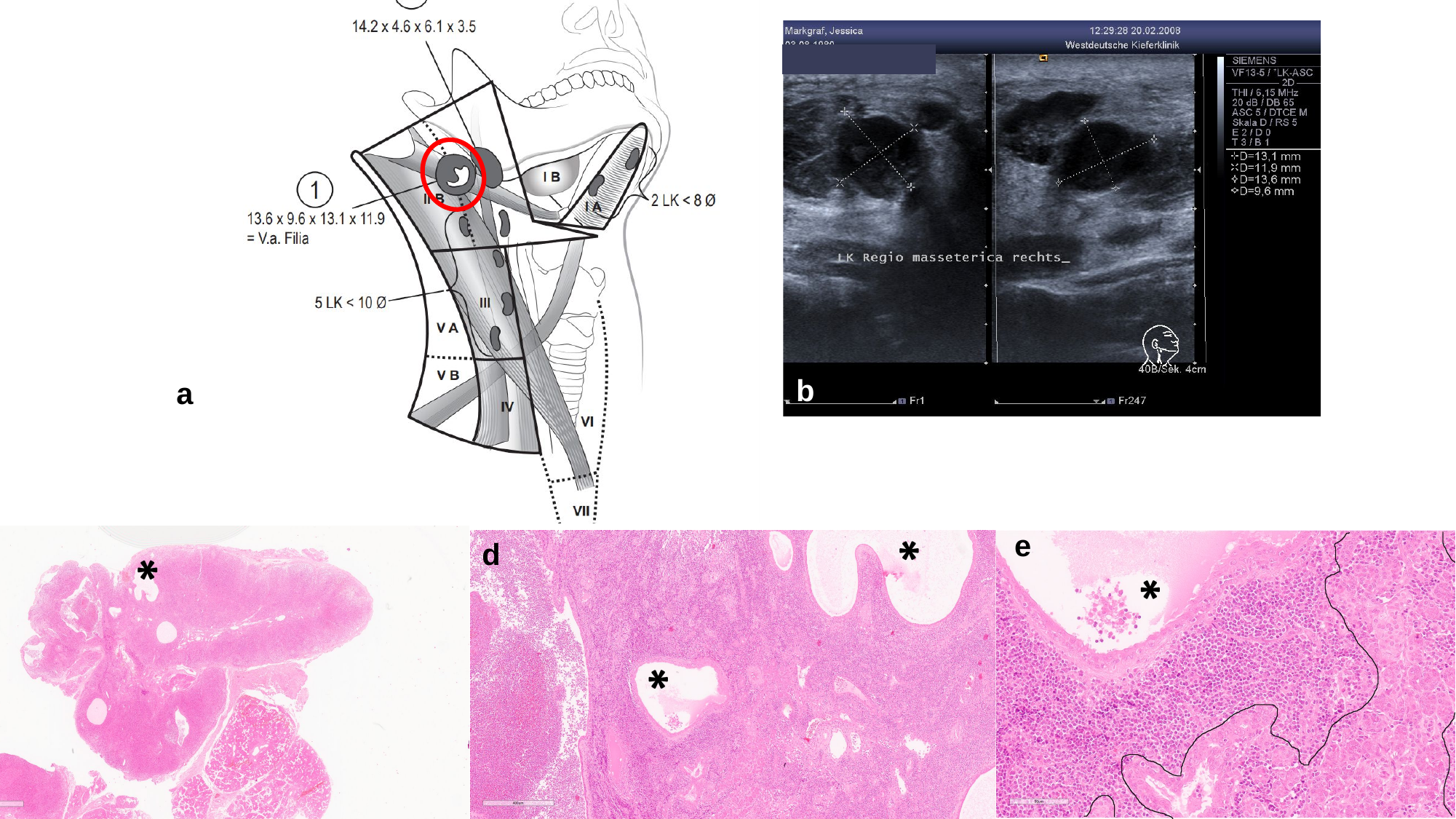

b
a
e
c
d

Supplement: Supplementary file 6 — Suppl. Figure 4: Representative false positive results. A. a: LN #1 of a 28-year-old female patient is shown. She reported a steadily increasing painless swelling for 2 weeks prior to the examination. b: Sonography showed two rounded LNs with a chaotic hypointense internal echo and clear pathological vascularization. The LN was classified as bearing a metastasis. The two indices (2D: 1.42, OCP for level IIA: 1.74 and 3D: 2.83, OCP for level IIA: 18.24) were also false positive. Histopathology revealed a LN with low-grade, chronic, unspecific lymphadenitis and salivary gland tissue heterotopia as well as adjacent other serous salivary gland parenchyma with cystic duct dilatation and no evidence of malignancy. c-e: The dilated gland lumina are indicated by asterisks. e: Gland acini and small ducts are indicated by black lines. Scale bars are presented in the lower left corners of the images, and the magnifications are as follows: 3 mm, 0.9x (c); 400 µm, 6.5x (d); and 90 µm, 24.4x (e). B. a: The level IIA lymph node shown was categorized as “probably filia” (counted as positive) in the subjective findings due to the spatial proximity to a metastasis in the bifurcation area and an eccentric cortical hypertrophy (red circles in b). The two indices (2D: 2.39, OCP for level IIA: 1.74 and 3D: 36.66, OCP for level IIA: 18.24) were correctly negative. c-e: Histopathology revealed a nonmetastatic lymph node with secondary follicles, and the tissue was examined at increasing magnifications. Representative germinal centers are indicated by asterisks. Scale bars are presented in the lower left corners of the images, and the magnifications are as follows: 2 mm, 1.3x (c); 300 µm, 7.8x (d); and 200 µm, 20x (e). C. a: The level III/IV LN shown was rated as suspicious in the subjective findings due to its spatial proximity to a metastasis in the bifurcation area and eccentric cortical hypertrophy (red circles in b). The LN was rated as negative for the evaluation. The two indices (2D: [file 432_2023_5439_MOESM6_ESM.pptx]

## Slide 1
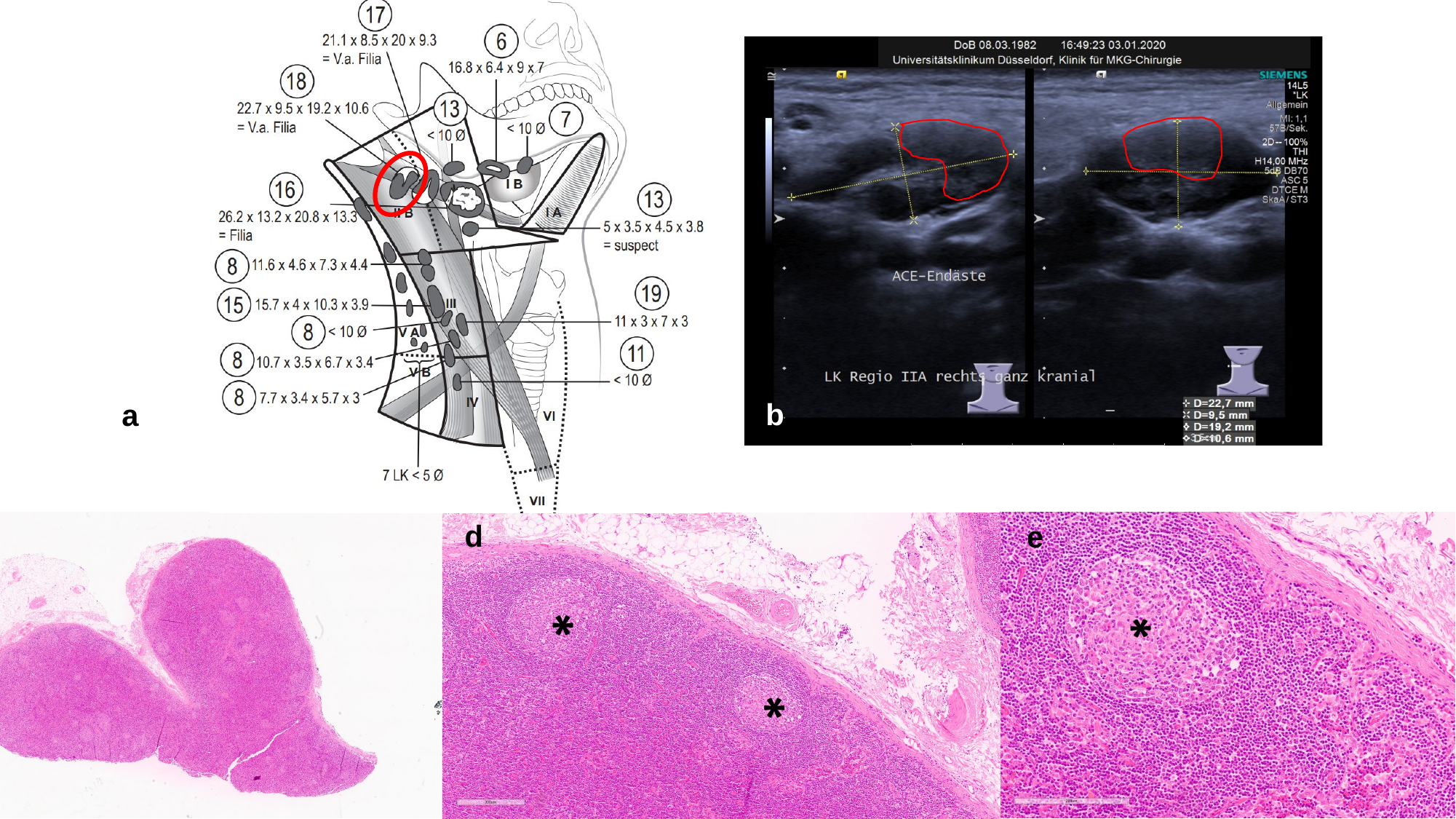

b
a
c
d
e

Supplement: Supplementary file 7 — Suppl. file7 (PPTX 14212 KB) [file 432_2023_5439_MOESM7_ESM.pptx]

## Slide 1
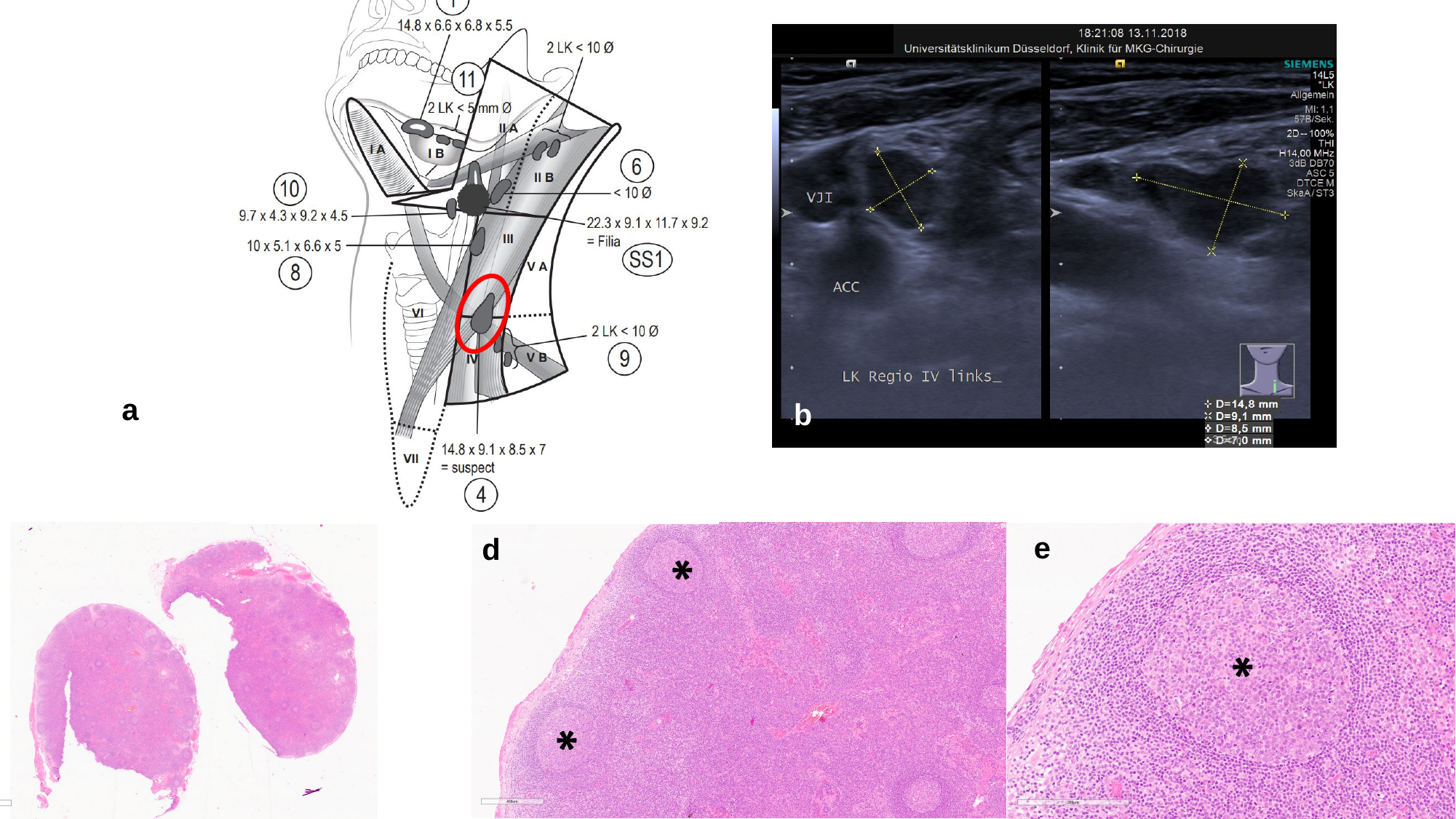

a
b
c
e
d

Supplement: Supplementary file 8 — Suppl. file8 (PPTX 11298 KB) [file 432_2023_5439_MOESM8_ESM.pptx]

## Slide 1
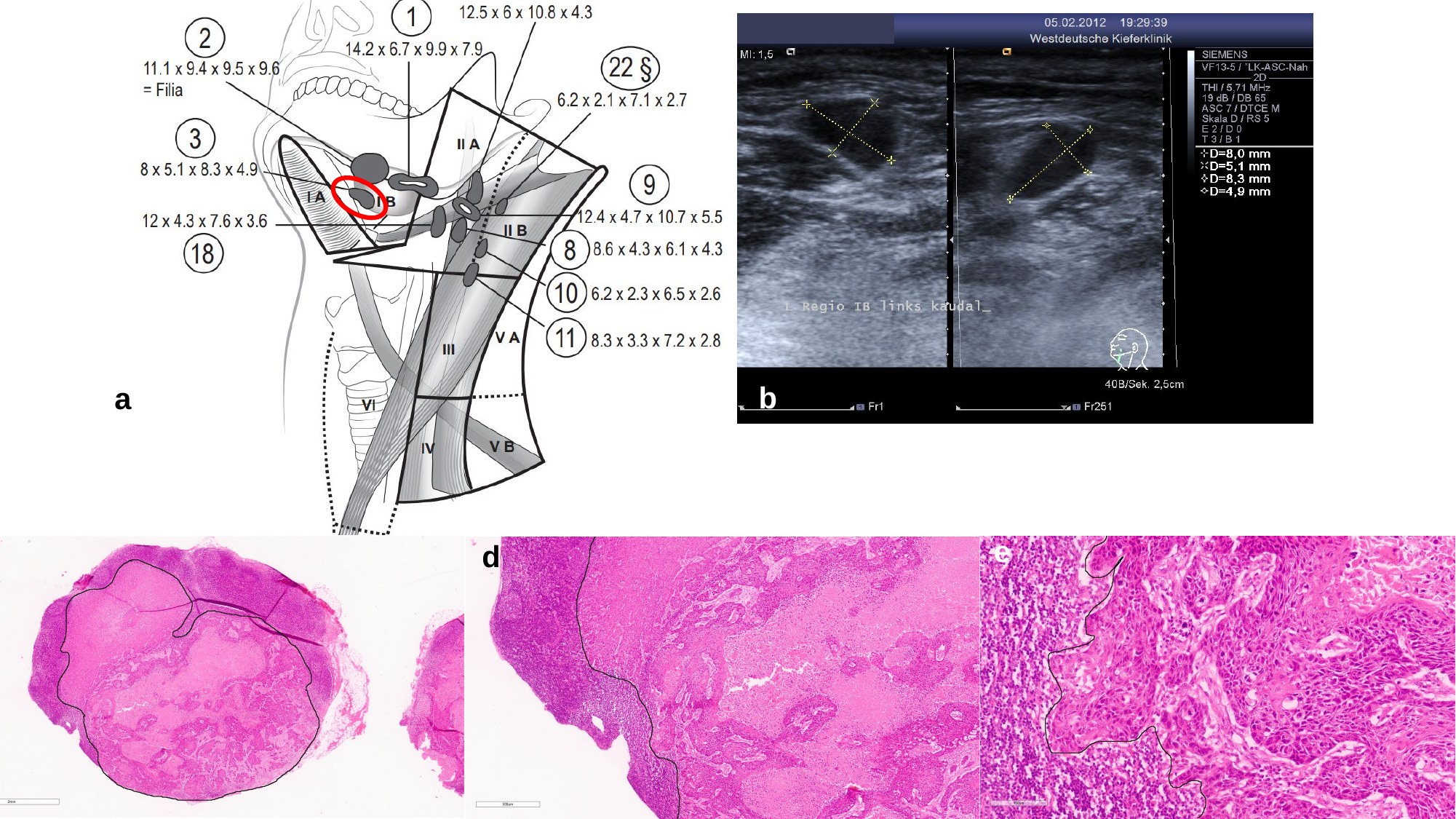

b
a
e
c
d

Supplement: Supplementary file 9 — Suppl. Figure 5: Representative false negative results: A. LN #3 of patient #136 was subjectively incorrectly classified as tumor free. Both the 2D roundness index of 1.69 (OCP for level IB: 1.61) and the 3D index of 8.18 (OCP for level IB: 7.03) incorrectly indicated "no metastasis". A metastasis of 6 x 4 mm in maximum diameter was found by histopathology. a: In the LN map, LN #3 is indicated by a red circle. b: B-scan of the LN in two perpendicular planes. c-e: Increasing magnifications of the LN metastasis with central necrosis of a squamous cell carcinoma. The borders of the metastasis are indicated by the black line, scale bars are presented in the lower left corners of the images, and the magnifications are as follows: 2 mm, 1.8x (c); 500 µm, 4.8x (d); and 100 µm, 21.2x (e). B. LN #18 of patient #132 was classified as metastasis-affected in the subjective ultrasound findings, as a small tumor with a maximum diameter of 7 mm was detected within the LN. Both the 2D index (2.94, OCP for level III: 2.01) and the 3D index (403.93, OCP for level III: 24.89) results were false negatives. The metastasis suspected in the B-scan was confirmed histopathologically (3 mm in maximum diameter). a: In the LN map, LN #18 is indicated by a red circle, b and c: B-scan of the LN in two perpendicular planes. The LN was measuring 18.8 x 6.4 x 8.2 x 6.3 mm and the metastasis within it 6.9 x 5.8 x 6.1 x 4.9 mm. d-f: Increasing magnifications of the LN containing a metastasis of nonkeratinizing squamous cell cancer, with increasing magnifications. The borders of metastasis are indicated by the black line, and scale bars are presented in the lower left corners of the images. The magnifications are as follows: 2 mm, 1.7x (d); 400 µm, 5.3x (e); and 90 µm, 23.2x (f). C. For LN #7 of patient #44, a small marginal finding of approximately 2 mm was detected in the B-scan, and this was not classified as a metastasis because of the hyperintense internal echo. The two indices (2D: 2.92, OCP in [file 432_2023_5439_MOESM9_ESM.pptx]

## Slide 1
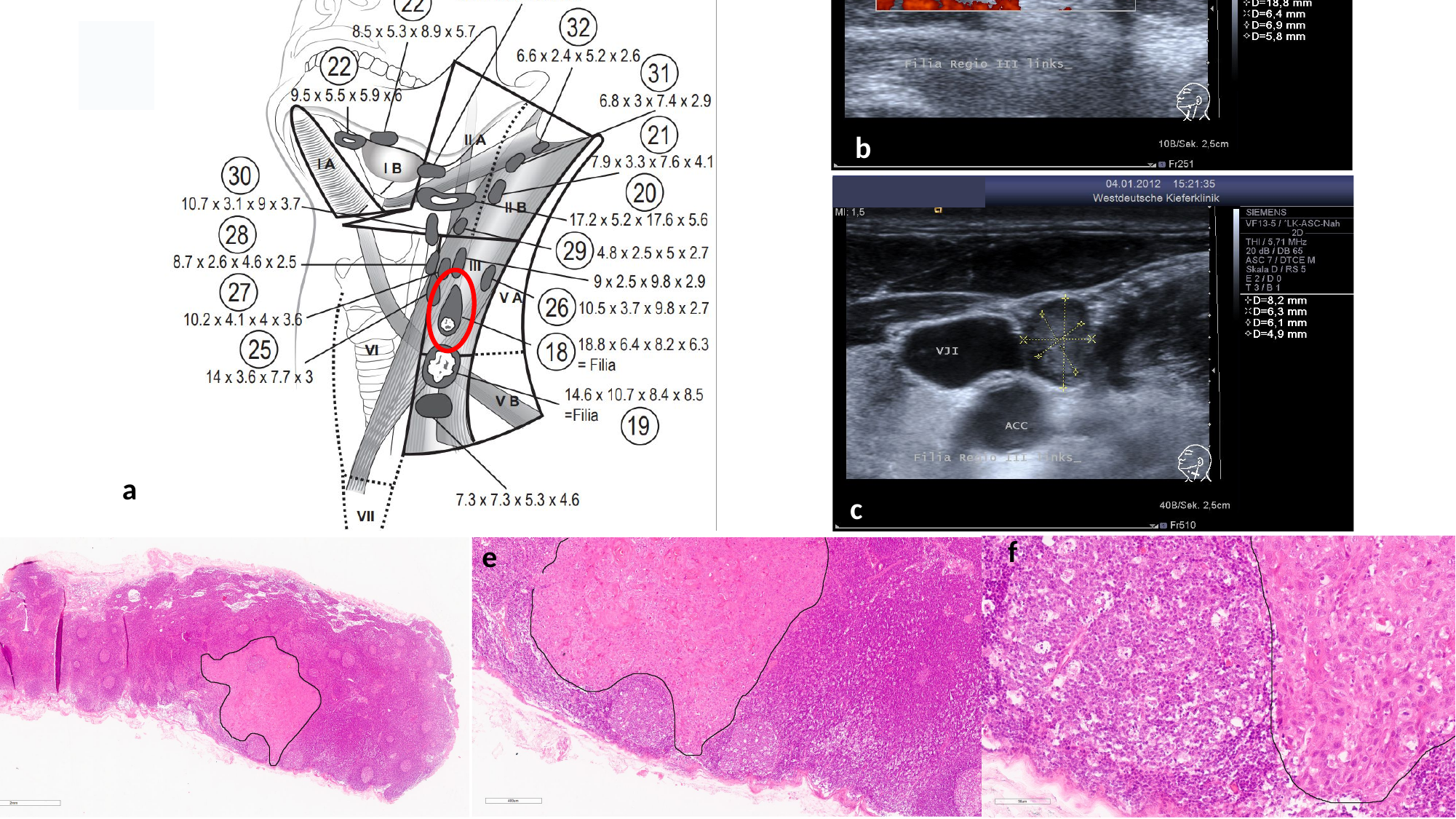

b
a
c
f
d
e

Supplement: Supplementary file 10 — Suppl. file10 (PPTX 14760 KB) [file 432_2023_5439_MOESM10_ESM.pptx]

## Slide 1
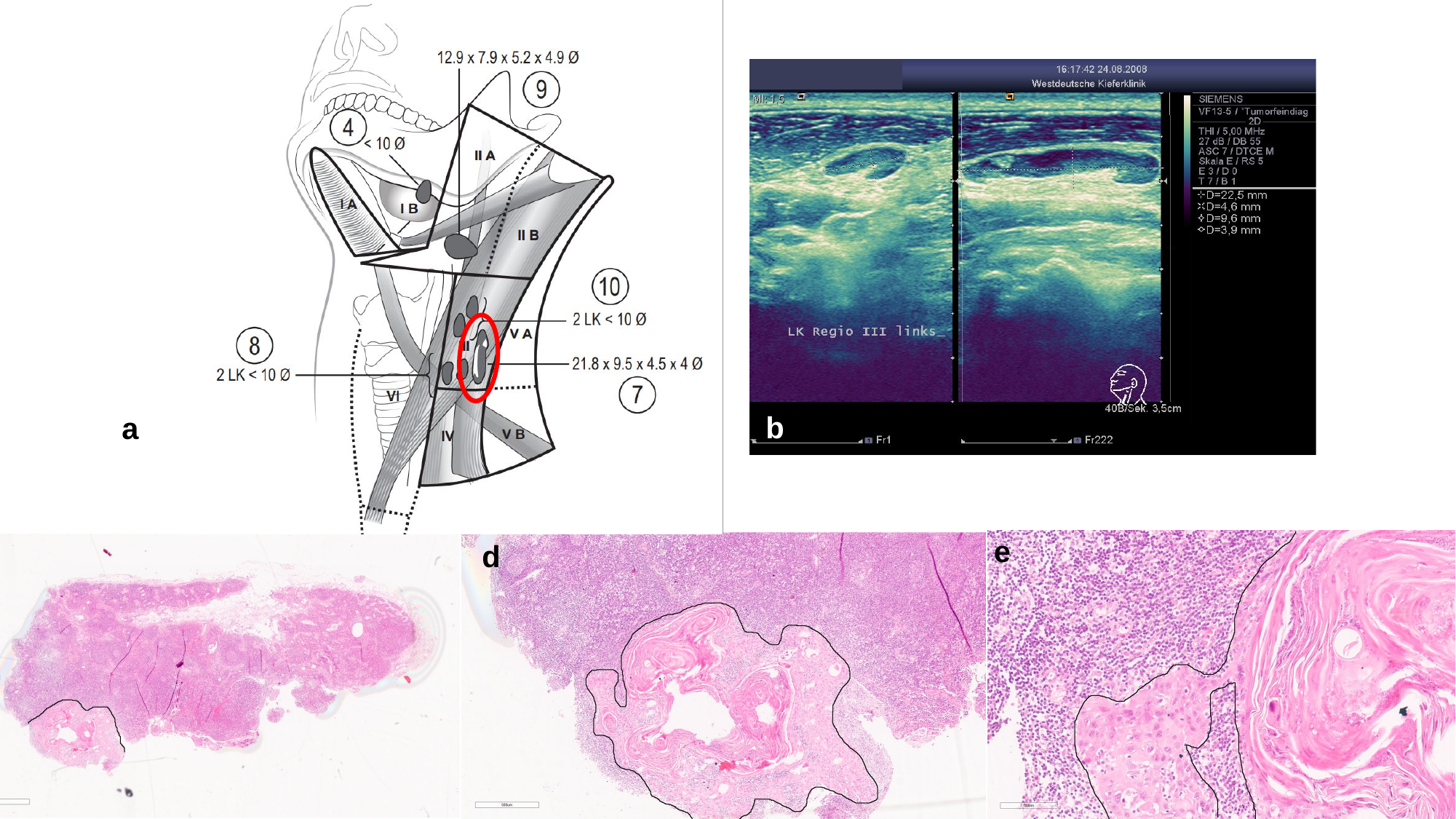

b
a
e
c
d

Supplement: Supplementary file 11 — Suppl. file11 (PPTX 13050 KB) [file 432_2023_5439_MOESM11_ESM.pptx]
