# Supplementary material for: Comparative analysis of diagnostic ultrasound and histopathology for detecting cervical lymph node metastases in head and neck cancer
Source: J Cancer Res Clin Oncol. 2023 Oct 12;149(19):17319–33. doi: 10.1007/s00432-023-05439-x (PMC10657327; doi:10.1007/s00432-023-05439-x)
Supplement: Supplementary file 5 — Suppl. Figure 3: CRF of the individually designed "Lymphsono" database based on the Omnicomm software (PDF 17 KB) [file 432_2023_5439_MOESM5_ESM.pdf]

Case Report Form Data

Program: KKSD/KKSD

Protocol : LYMPHSONO/DE (Lymphknoten-Sonographie Register)

Date: 30.07.2015 12:43:59 CEST

User: SPROLL

From Patient ID : To Patient ID :

Program: KKSD/KKSD **Protocol :** LYMPHSONO/DE
Center : DDORF **Patient ID :** 225
Event ID : EE **Page ID :** Operation (Ersterkrankung)
Page Entry Status: V

-- Module OP (Operation (Ersterkrankung)) --

Geburtsdatum 20.06.1951

Geschlecht ☑ männlich ☐ weiblich

Frühere maligne Tumoren

- ☑ 0-nein
- ☐ 1-Kopf-Hals-Karzinom
- ☐ 2-Verdauungstrakt
- ☐ 3-Lunge, Pleura
- ☐ 4-Knochen, Weichteile
- ☐ 5-Haut
- ☐ 6-Brust
- ☐ 7-Gynäkologische Tumoren
- ☐ 8-Urologische Tumoren
- ☐ 9-Nebennierenrinden-Tumoren
- ☐ 10-Ophthalmologische Tumoren
- ☐ 11-Morbus Hodgkin
- ☐ 12-NHL

Datum der Ultraschalluntersuchung präoperativ 30.01.2015

.		Anzahl der untersuchten LK	davon positiv
**(r)uT-Stadium allein aufgrund der Ultraschalluntersuchung**	T1 T1		
**(r)uN-Stadium allein aufgrund des Solbiati-Index**	N1 N1	8	1
**(r)uN-Stadium allein aufgrund des Roundness-Index**	N0 N0	8	0
**(r)uN-Stadium aufgrund des gesamten Sonobefundes**	N0 N0	16	0

OP-Datum 11.02.2015

Art der Tumoroperation (Teilresektion)

- ☐ 1-Mundboden
- ☐ 2-Zunge
- ☐ 3-Unterkiefer
- ☐ 4-Oberkiefer
- ☐ 5-Wange(n)
- ☐ 6-Vestibulum
- ☐ 7-Lippen
- ☐ 8-Weichgaumen
- ☐ 9-Mundboden und Zunge
- ☐ 10-Mundboden, Unterkiefer und Vestibulum
- ☐ 11-Mundboden und Unterkiefer
- ☑ 12-Mundboden, Unterkiefer und Wange(n)
- ☐ 13-Mundboden, Zunge und Unterkiefer
- ☐ 14-Lippen und Vestibulum
- ☐ 15-Lippen, Vestibulum und Unterkiefer
- ☐ 16-Lippen, Vestibulum und Oberkiefer
- ☐ 17-Oberkiefer und Wange(n)
- ☐ 18-Unterkiefer und Wange(n)
- ☐ 19-Oberkiefer und Weichgaumen
- ☐ 20-Oberkiefer, Pharynx und Unterkiefer
- ☐ 21-Weichgaumen und Pharynx
- ☐ 22-Skalp
- ☐ 23-Oberlid
- ☐ 24-Unterlid
- ☐ 25-Ohr
- ☐ 26-Nase
- ☐ 27-Nase, Weich- und Hartgaumen
- ☐ 28-Orbita
- ☐ 29-Hypopharynx
- ☐ 30-Oropharynx
- ☐ 31-Tonsille

Seite der Tumoroperation

☑ links ☐ rechts ☐ beidseits

Art der Lymphknotenausräumung (links)

- ☐ Radikale Neck Dissection
- ☐ Konservierende Neck Dissection
- ☐ Selektive Neck Dissection

**Ia**	☑ .	**III**	☑ .	**VI**	☐ .
**Ib**	☑ .	**IV**	☐ .	**VII**	☐ .
**IIa**	☑ .	**Va**	☐ .		
**IIb**	☑ .	**Vb**	☐ .		

Completed: ***Date:***

Program: KKSD/KKSD **Protocol :** LYMPHSONO/DE
Center : DDORF **Patient ID :** 225
Event ID : EE **Page ID :** Operation (Ersterkrankung)
Page Entry Status: V

Ausräumung

- ☐ 1-Bukzinator
- ☐ 2-periparotideal
- ☐ 3-parotideal
- ☐ 4-retroaurikulär
- ☐ 5-subokzipital
- ☐ 6-infraklavikulär
- ☐ 7-Axilla Regio I
- ☐ 8-Axilla Regio II
- ☐ 9-Axilla Regio III
- ☐ 10-mediastinal

Art der Lymphknotenausräumung (rechts)

- ☐ Radikale Neck Dissection
- ☐ Konservierende Neck Dissection
- ☐ Selektive Neck Dissection

Ia ☐ . **III** ☐ . **VI** ☐ .
Ib ☐ . **IV** ☐ . **VII** ☐ .
IIa ☐ . **Va** ☐ .
IIb ☐ . **Vb** ☐ .

Ausräumung

- ☐ 1-Bukzinator
- ☐ 2-periparotideal
- ☐ 3-parotideal
- ☐ 4-retroaurikulär
- ☐ 5-subokzipital
- ☐ 6-infraklavikulär
- ☐ 7-Axilla Regio I
- ☐ 8-Axilla Regio II
- ☐ 9-Axilla Regio III
- ☐ 10-mediastinal

pT-Stadium T1 T1
pN-Stadium N0 N0
Anzahl der histopathologisch untersuchten Lymphknoten 8
davon positiv 0
M-Stadium pM0 pM0
L-Stadium L0 L0
V-Stadium V0 V0
Pn-Stadium Pn0 Pn0
G-Stadium G2 G2
R-Stadium R0 R0

Primärtumor vorhanden? ☑ ja ☐ nein
Länge des Tumors in mm 19
Breite des Tumors in mm 15
Dicke des Tumors in mm
Topographischer ICD-O-Code des Primärtumors C03.1L
Morphologischer ICD-O-Code des Primärtumors 8071/3

Übereinstimmung Solbiati-Index vs. pN-Stadium

- ☐ ja
- ☑ überschätzt
- ☐ unterschätzt

positive LK 1
histopathologisch positive LK 0
falsch positive LK 1
falsch negative LK 0
Differierende LK 1
Richtige Entscheidung aufgrund einer Fehldiagnose ☐ ja ☑ nein

Übereinstimmung Roundness-Index vs. pN-Stadium

- ☑ ja
- ☐ überschätzt
- ☐ unterschätzt

positive LK 0
histopathologisch positive LK 0
falsch positive LK 0
falsch negative LK 0
Differierende LK 0
Richtige Entscheidung aufgrund einer Fehldiagnose ☐ ja ☑ nein

Completed: *Date:*

Program: KKSD/KKSD **Protocol :** LYMPHSONO/DE
Center : DDORF **Patient ID :** 225
Event ID : EE **Page ID :** Operation (Ersterkrankung)
Page Entry Status: V

Übereinstimmung Sonobefund subjektiv vs. pN-Stadium

- ☑ ja
- ☐ überschätzt
- ☐ unterschätzt

**positive LK**	0
**histopathologisch positive LK**	0
**falsch positive LK**	0
**falsch negative LK**	0
**Differierende LK**	0

Richtige Entscheidung aufgrund einer Fehldiagnose ☐ ja ☑ nein

Übereinstimmung Solbiati-Index vs. pN0/pN+-Stadium

- ☐ ja
- ☑ überschätzt
- ☐ unterschätzt

Richtige Entscheidung aufgrund einer Fehldiagnose ☐ ja ☑ nein

Übereinstimmung Roundness-Index vs. pN0/pN+-Stadium

- ☑ ja
- ☐ überschätzt
- ☐ unterschätzt

Richtige Entscheidung aufgrund einer Fehldiagnose ☐ ja ☑ nein

Übereinstimmung Sonobefund subjektiv vs. pN0/pN+-Stadium

- ☑ ja
- ☐ überschätzt
- ☐ unterschätzt

Richtige Entscheidung aufgrund einer Fehldiagnose ☐ ja ☑ nein

Bestrahlung

Primarius ☑ ja ☐ nein **ad [Gy]** 66

Lymphabflußwege ☐ ja ☑ nein **links ad [Gy]** **rechts ad [Gy]**

Chemotherapie ☐ ja ☑ nein

welche

- ☐ 2 Zyklen 5-FU, Cisplatin
- ☐ Docetaxel + Cisplatin
- ☐ ACCRA-HN (Cetuximab,Cispt,5-FU,adjuv.)
- ☐ HART-A (5-FU, Mitomycin)
- ☐ HART-B (5-FU, Cisplatin)
- ☐ andere

Completed: ***Date:***
