# Supplementary material for: Comparative analysis of diagnostic ultrasound and histopathology for detecting cervical lymph node metastases in head and neck cancer
Source: J Cancer Res Clin Oncol. 2023 Oct 12;149(19):17319–33. doi: 10.1007/s00432-023-05439-x (PMC10657327; doi:10.1007/s00432-023-05439-x)
Supplement: Supplementary file 12 — Suppl. Table 1: Excerpt from the primary database for an overview of the individual lymph nodes seen as false negatives in the subjective US examination that were responsible for upstaging from cN0 to cN+ (PDF 24 KB) [file 432_2023_5439_MOESM12_ESM.pdf]

Patient	Submission number in pathology	Location of the LN (AAO-AHNS LN level)	Specimen No.	LN size in Ultrasound (lxbxd1xd2 in mm)	Assessment based on B-scan ultrasonography (0=negative; 1=positive).	Histology (0= negative, 1 =positive)	LN size in Histology (mm)	Metastasis size in Histology (mm)	cN stage (based on the subjective ultrasound findings)	pN-stage	Correspondence sono-findings vs. patho-findings 0 = yes; 1 = overestimated; 2 = underestimated.	Remarks
5	H ...-07	IB right ventral	5§	8,4x7,3x7,0x6,7	**0**	**1**	6x4	2,6x1,3	cN0(0/9)	**pN1(1/21)**	2	
22	H ...-07	IIA right	1	29x18,2x8,7x8,1	**0**	**1**	10x8	3,8x2,7	cN0(0/9)	**pN1(1/11)**	2	
36	H ...-08	IB left ventral	1	12,3x9,5x7,2x6,6	**0**	**1**	4,5x3	3,2x2,6	cN0(0/17)	**pN1(1/19)**	2	
44	H ...-08	III left	7	21,8x9,5x4,5x4	**0**	**1**	10x4,5	2,2x1,7	cN0(0/10)	**pN1(1/36)**	2	
59	H ...-09	IIA right	1	29x20,3x9x7,9	**0**	**1**	8x6	6x4	cN0(0/17)	**pN2c(1/50)**	2	
66	H ...-09	IIA right	2§	19,2x15,1x5,9x5,8	**0**	**1**	18x5	5x4	cN0(0/16)	**pN1(1/19)**	2	
69	H ...-09	III left caudal	6	19,5x9,3x5,4x3,6	**0**	**1**	7x4	5x3,5	cN0(0/17)	**pN2c(1/76)**	2	
70	H ...-09, H ...-09, H ...-09	IIA left cranial	11	22,2x18,5x5,8x5,7	**0**	**1**	11x7	5,5x4	cN0(0/23)	**pN2b(2/108)**	2	
		IIA right cranial	V	22,9x17,3x5,8x5,7	**0**	**1**	20x12	2,9x1,6				
97	H ...-10	IB right ventral	2	9,8x7,5x4x3,7	**0**	**1**	6x4,5	5x3,5	cN0(0/25)	**pN2b(3/9)**	2	
		IB right centered	3	10,2x9,1x7,5x7,2	**0**	**1**	7x5	1,6x0,8				
		IB right dorsal	4	8,2x7,2x5,7x5,3	**0**	**1**	ND	ND				
108	H ...-11	IB left dorsal	4	13x8,3x4,6x3,5	**0**	**1**	5x5	0,9x0,7	cN0(0/15)	**pN1(1/20)**	2	
111	H ...-11	IIA left	2	26,1x13,2x12,2x10,8	**0**	**1**	14,5x7	1,6x1,5	cN0(0/20)	**pN1(1/41) (mi)**	2	
116	H ...-11								cN0(0/27)	**pN1(1/32)**	2	Metastasis in LN that was not described in the ultrasound findi
118	H ...-11	IB right dorsal	10	8,2x3,9x7,5x3,4	**0**	**1**	4,5x4,5	4,4x2	cN0(0/25)	**pN2c(3/26)**	2	
		III left caudal	12	13,4x4,3x11,8x4,4	**0**	**1**	9x9	6,5x3,5				
123	H ...-11	IIA right ventral	19	15,7x5,2x10,9x4,4	**0**	**1**	14x8	8x5	cN0(0/30)	**pN1(1/39)**	2	
124	H ...-11	IIA left caudal	2	19,8x7,2x19,5x6,5	**0**	**1**	20x14	4x1,5	cN0(0/24)	**pN2c(3/30)**	2	
		IB right dorsal	20	9,6x5x8,8x5,2	**0**	**1**	17x9	2x1,5				
		IIA right caudal	21	16,6x6,3x13,7x4,6	**0**	**1**	10,5x7	1,5x1				
161	H ...-12	IIA left ventral	11	8x4,6x7,8x4,4	**0**	**1**	11x6	4x,2,5	cN0(0/17)	**pN1(1/47)**	2	
165	H ...-13	III left kraniodorsal	21	12,4x3,4x12,4x4	**0**	**1**	9x5	1,5x1	cN0(0/19)	**pN1(1/26; mi)**	2	
166	H ...-13	III left caudal	4	17,2x4,7x8,2x3,9	**0**	**1**	6x3	3x1	cN0(0/22)	**pN1(1/48)**	2	
193	H ...-13	IB left ventral	1	15,8x6,4x14,4x6,5	**0**	**1**	12x8	6x5	cN0(0/18)	**pN1(1/32)**	2	
194	H ...-13	IIA right	4	14,7x3,9x12,4x3,7	**0**	**1**	13x10	3x1	cN0(0/11)	**pN1(1/22)**	2	
201	H ...-14	IIA right caudal	1	19,3x8,2x14,7x6,7	**0**	**1**	7x5	0,3x0,3	cN0(0/20)	**pN1(1(mi)/25)**	2	
211	H ...-14								cN0(0/19)	**pN1(1/23)**	2	Metastasis in LN that was not described in the ultrasound findi
217	H ...-14	IB left dorsal	4	10x6,3x9,7x6,2	**0**	**1**	16x7	6x5	cN0(0/25)	**pN1(1/28)**	2	
